# Satellite cell heterogeneity revealed by G-Tool, an open algorithm to quantify myogenesis through colony-forming assays

**DOI:** 10.1186/2044-5040-2-13

**Published:** 2012-06-15

**Authors:** Joseph Ippolito, Robert W Arpke, Kerri T Haider, Jianyi Zhang, Michael Kyba

**Affiliations:** 1 Lillehei Heart Institute, University of Minnesota, Minneapolis, MN, USA; 2 Department of Medicine, University of Minnesota, Minneapolis, MN, USA; 3 Bioengineering Program, University of Minnesota, Minneapolis, MN, USA; 4 Department of Pediatrics, University of Minnesota, Minneapolis, MN, USA; 5 Nils Hasselmo Hall, 312 Church St. S.E., Minneapolis, MN, USA

**Keywords:** Colony assay, Diaphragm, Image analysis, Myogenesis, Oxygen, Pax7, Satellite cells

## Abstract

**Background:**

Muscle growth and repair is accomplished by the satellite cell pool, a self-renewing population of myogenic progenitors. Functional heterogeneity within the satellite cell compartment and changes in potential with experimental intervention can be revealed by *in vitro* colony-forming cell (CFC) assays, however large numbers of colonies need to be assayed to give meaningful data, and manually quantifying nuclei and scoring markers of differentiation is experimentally limiting.

**Methods:**

We present G-Tool, a multiplatform (Java) open-source algorithm that analyzes an ensemble of fluorescent micrographs of satellite cell-derived colonies to provide quantitative and statistically meaningful metrics of myogenic potential, including proliferation capacity and propensity to differentiate.

**Results:**

We demonstrate the utility of G-Tool in two applications: first, we quantify the response of satellite cells to oxygen concentration. Compared to 3% oxygen which approximates tissue levels, we find that 21% oxygen, the ambient level, markedly limits the proliferative potential of transit amplifying progeny but at the same time inhibits the rate of terminal myogenic differentiation. We also test whether satellite cells from different muscles have intrinsic differences that can be read out *in vitro*. Compared to masseter, dorsi, forelimb and hindlimb muscles, we find that the diaphragm satellite cells have significantly increased proliferative potential and a reduced propensity to spontaneously differentiate. These features may be related to the unique always-active status of the diaphragm.

**Conclusions:**

G-Tool facilitates consistent and reproducible CFC analysis between experiments and individuals. It is released under an open-source license that enables further development by interested members of the community.

## Background

Muscle wasting associated with aging, cancer, and muscular dystrophy, is a grave health problem affecting millions of people worldwide. Understanding the physiological regulation of muscle growth and regeneration is therefore an important current goal in the field of skeletal muscle research. Likewise, understanding how cells respond to *ex vivo* culture, and developing optimized conditions for expansion of myogenic progenitors, could have a significant impact if this knowledge can be applied to myogenic cell transplantation. Skeletal muscle growth and regeneration is mediated by the satellite cell, a cell situated adjacent to and beneath the basal lamina of the myofiber [1]. When satellite cells are transplanted into injured muscle, they give rise to new muscle fibers, in addition to contributing to the satellite cell pool of the recipient muscle [2-4]. However, when cultured *in vitro*, their transit-amplifying progeny rapidly lose muscle engraftment capability [4]. Accordingly, trials involving transplantation of human myoblast cultures for the treatment of Duchenne muscular dystrophy ended in failure [5,6]. Direct transplantation is not feasible because harvest of satellite cells from muscle tissue results in substantial damage to the donor, so only small numbers of satellite cells can be obtained, insufficient for therapeutic application. However, single satellite cells clearly have the intrinsic potential to self-renew and generate satellite cell progeny *in vivo*[3], so it remains possible that appropriate culture conditions may enable expansion of repopulating activity, and thereby enable cell therapies. Finding optimal culture conditions is therefore a near-term goal of considerable importance.

The colony-forming cell (CFC) assay has had a tremendous impact in the field of hematopoiesis, both on our understanding of the hematopoietic stem cell (HSC), and of its transit amplifying progeny. The key cytokines that regulate the HSC, stem cell factor (SCF) and thrombopoietin (TPO), were both identified through CFC assays [7-9]. Because the transit-amplifying progeny of the HSC diverge into multiple lineages, a multitude of hematopoietic colony types exist, from unilineage (for example, erythroid or macrophage) to multilineage (for example, granulocyte-erythrocyte-macrophage-megakaryocyte (GEMM)), and their different cellular constitution allows rapid CFC evaluation. Hematopoietic colonies can thus be classified within seconds simply by scoring colony morphology visually. However, colonies derived from the unilineage stem cell of the skeletal muscle look superficially similar: they are composed of a mixture of mononuclear cells and fused myotubes. Therefore, gleaning information about the state of the cell or its response to the culture condition requires analysis of the cellular composition of the colony, for example determining how many total cells the colony has (a measure of proliferative potential of the CFC), and the ratio of undifferentiated to terminally differentiated cells (a measure of propensity to differentiate). Immunostaining, photographing many colonies, manually counting nuclei and determining fusion indices for each can provide this useful information, but application of this approach is limited by the time-consuming nature of manual inspection of colonies with hundreds of cells. An automated way of processing this information would make the CFC assay much more accessible to the analysis of skeletal muscle progenitors.

We describe here G-Tool, an open source Java algorithm with a simple graphical user interface (GUI), that allows the evaluation of an ensemble of colony photographs and provides quantitative metrics of proliferation and differentiation potential for each CFC and statistical summaries allowing different experimental groups to be compared for differences in CFC activity. We then apply G-Tool to two biological questions: measuring the effect of oxygen on CFC activity, and determining whether satellite cells from different muscle groups have intrinsic differences in proliferation and differentiation potential.

## Methods

### Satellite cell isolation

Satellite cells were isolated from Pax7-ZsGreen mice [10], which were housed in a pathogen-free barrier facility and cared for with the oversight of the University of Minnesota Institutional Animal Care and Use Committee (protocol #1107A02663). Briefly, hind limb muscle was removed. With a razor blade parallel to the muscle fiber, forceps were used to separate the fibers. Muscle was incubated shaking with 0.2% collagenase type II (Gibco, Grand Island, NY; 17101-015) in DMEM/high glucose medium containing 4.00 mM l-glutamine, 4,500 mg/l glucose, and sodium pyruvate (HyClone, Logan, UT; SH30243.01) at 37°C for 75 minutes. The sample was washed two times with rinsing solution (F-10+), Ham’s/F-10 medium (HyClone, SH30025.01) supplemented with 10% horse serum (Gibco, 26050-088), 1% 1 M HEPES buffer solution (Gibco, 15630-106), and 1% penicillin/streptomycin (Gibco, 15140-122). Muscle samples were poured into a petri dish and mechanically scraped with a sheared Pasteur pipette. The sample was centrifuged and washed again with F-10+. The sample was resuspended in F-10+ containing collagenase II and dispase (Gibco, 17105-041), vortexed and incubated shaking at 37°C for 30 minutes. After incubation, the sample was again vortexed. The sample was drawn and released four times with a 16-gauge needle, then with an 18-gauge needle to dislodge cells from the muscle fibers before applying the sample to a 40 μm cell strainer. The cell suspension was centrifuged at 1,500 RPM for 5 minutes, resuspended in F-10+, drawn and released four times again with an 18-gauge needle and applied to a new 40 μm cell strainer. After centrifugation, the sample was resuspended in fluorescence-activated cell sorting (FACS) staining medium: phosphate-buffered saline (PBS) (HyClone, SH30256.01) containing 2% fetal bovine serum (FBS; HyClone).

### Cell culture, staining, and imaging

Satellite cells were identified by FACS and single cells were deposited into 96-well plates in myogenic medium: DMEM/F12 medium without l-glutamine (Cell Gro, Manassas, VA; 15-090-CV) containing 20% FBS (HyClone), 10% horse serum (Gibco, 26050-088), 50 ng/μL human basic fibroblast growth factor (Peprotech, Rocky Hill, NJ; 100-18), 1% penicillin/streptomycin (Gibco, 15140-122), 1% Glutamax (Gibco, 35050-061), and 0.5% chick embryonic extract (US Biological, Swampscott, MA; C3999).

Plates were grown at 37°C in either normal tissue culture incubators (at 5% CO_2_) or under reduced oxygen conditions using a glass chamber filled with a custom gas mixture (3% O_2_, 5% CO_2_, 92% N_2_). The chamber was then sealed and maintained for 8 days at 37°C. On day 8 plates were removed from their respective incubators and colonies were identified and fixed with 4% paraformaldehyde for 20 minutes at room temperature. Colonies were permeablized with 0.3% triton-X for 20 minutes at room temperature, washed one time with PBS, and blocked with 3% bovine serum albumin (BSA) in PBS for 1 h at room temperature. Colonies were stained at 4°C with MF 20, a monoclonal antibody against sarcomeric myosin (obtained from the Developmental Studies Hybridoma Bank, University of Iowa), at a dilution of 1:20 in 3% BSA in PBS overnight at 4°C. The following day, plates were washed three times with PBS and incubated with a 1:500 dilution of Alexa Fluor 555 goat anti-mouse secondary antibody (Invitrogen, Grand Island, NY; A21422) for 45 minutes at room temperature. After three washes with PBS, colonies were then counterstained with a 1:1,000 dilution of 4',6-diamidino-2-phenylindole (DAPI) in PBS, washed one final time with PBS. The stained cells were covered with PBS and imaged on a Zeiss AxioObserver Z1 inverted microscope with an AxioCamMR3 camera (Thornwood, NY).

### G-tool GUI and algorithm design

The G-Tool GUI was written using the NetBeans open-source JAVA Integrated Development Environment (Redwood Shores, CA). The cell counting algorithm which performs the processing was written using MATLAB R2011a, an engineering programming language from MathWorks (Natick, MA, USA), and requires the end user to install the MATLAB runtime libraries found within the MATLAB Runtime Compiler (MCR) library package.

### Defining and counting nuclear areas

Once an image is passed into the algorithm, the removal of background noise is performed by applying a flat, disk-shaped, structuring element with a user defined radius, R, to the image using the MATLAB function ‘imopen(img,strel(‘disk’,R)’. Image contrast modification is performed using a simple thresholding value, T. Thresholding was originally performed using Otsu’s Method, but this was found to be less accurate for our library of stained images than simple value thresholding. The modification of the structuring element’s radius and the thresholding value is performed through the settings tab of the G-Tool GUI, and both of these values can be set independently for each stain selected. The algorithm performs the nuclear counting using only the blue channel isolated from the merged RGB image, which accurately represents the DAPI stained nuclei. Maximization in image contrast allows for more accurate delineation between nuclei in the image. Computations performed on a single color channel in MATLAB represent that channel as a black and white image. Taking advantage of the black and white nature of this single-color-channel computing in MATLAB, the IPT function ‘BWBOUNDARIES’ allows for the identification of distinct regions in the image, which for cellular images in the blue channel represent nuclei. The centroid, or geometric centerpoint, is then calculated for each DAPI + region.

The algorithm then performs a dataset-wide calibration of the counting algorithm by creating a histogram of sizes for every distinct region found by the ‘BWBOUNDARIES’ function from the blue channel of every image. For satellite cell colonies, the first or largest peak on the resulting histogram typically represents the DAPI + regions that are single nuclei. The mean value of the DAPI + areas from within the first peak is then calculated. Centroids for regions within the first peak are arbitrarily given a nuclear quantity of 1. Every region size that is outside of the boundaries of the first peak is then divided by the first peak mean value, yielding the value for the number of nuclei represented by each nuclear centroid outside of the first peak. Ideally, the size of the smallest and largest single nucleus should represent the lower and upper bound of the first peak of the histogram, respectively. We have found that presenting the nuclear size data to the user as a histogram and asking the user to select the limits for lower bound and upper bound of the ‘first peak’ actually shortcuts having to manually analyze the algorithm’s rasterized output to determine whether single nuclei are being correctly identified. This more tedious calibration can still be performed by visual inspection if the user prefers to not use the histogram calibration method, or if the dataset in question encounters some unexpected issues. The histogram calibration method begins with the user inputting starting values for R and T and then proceeds with an iterative calibration of the dataset until he or she is satisfied that the values chosen for R and T best apply to the dataset being scanned. Inputting larger R and T values can help separate nuclei on images that have fuzzy nuclear boundaries.

### Defining myosin heavy chain (MHC) + regions

The second part of the algorithm applies a modified nuclear counting technique to determine the regions of the images that have stained positive for MHC. The MHC primary antibody was detected with an Alexa Fluor 555-conjugated secondary antibody. Regions staining MHC + appear in the red channel of a merged RGB image, with little to no bleeding into the other two channels. After isolation of the red color channel, noise is removed using the aforementioned noise/background removal technique. The red channel is then fed into the ‘BWBOUNDARIES’ function, whose ‘regions’ output corresponds to the perimeter boundaries of the MHC + areas. For MHC + datasets that appear dimly stained, decreasing the threshold value, T, for the red channel will allow for greater sensitivity.

### Quantifying nuclei per MHC + region

The number of nuclei that lie within MHC + regions is determined by mapping the locations of the DAPI+/nuclear centroids to the red MHC channel. Using the calibrated mean area value for a single nucleus allows determination of the number of nuclei within each nuclear region represented by a centroid. Mean area values for each centroid mapping to a given MHC + region are added. Their sum is designated as the probabilistic estimation of number of nuclei in a given MHC + region. The algorithm cycles through each region and performs this calculation across the entire image. The data are then parsed and displayed for the user through the GUI. This data is also autosaved to a comma separated value (CSV) file for convenience.

The algorithm processes one image at a time and appends the data from each successive image to a Microsoft Excel-compatible CSV file, and displays that data on a chart visible within the GUI.

## Results

### The satellite cell CFC assay

Within adult muscle, satellite cells are specifically marked by expression of the transcription factor Pax7 [11]. We isolated satellite cells from a BAC transgenic fluorescent reporter strain, Pax7-ZsGreen [10], single cell-sorted them into 96-well dishes, and allowed colonies to develop for 8 days. Single cell-derived colonies that arise in this assay are comprised of a mixture of single cells and fused myotubes, each colony having a unique size and level of differentiation. By further staining for sarcomeric MHC, single cells can be separated into less differentiated (MHC-) and more differentiated (MHC+) fractions, and fusion products can be readily identified as MHC + cells with three or more nuclei. By inspection of DAPI/MHC stained colonies, several CFC parameters can be defined, including total number of nuclei, a measure of proliferative potential; frequency of nuclei within MHC + cytoplasm, a measure of differentiation, and fusion index, that is, frequency of nuclei within MHC + myotubes, a measure of terminal differentiation.

### The G-tool algorithm

As these metrics are variable from colony to colony, large numbers of colonies must be evaluated to identify differences between two experimental groups. To facilitate this, we developed G-Tool, an algorithm that inspects an image of a single colony and assigns values to each metric. G-Tool is managed by a graphical user interface (GUI) that allows one to modify scanning parameters to offset the effects that image and staining quality may have on the CFC assay. The GUI is pointed to a specified directory, batch processes all of the image files within, rasterizes each image, outputs results for each colony, and produces a statistical summary for the group. The source code for both the GUI and the algorithm can be found in the supplemental information ( Additional file 1: G-Tool Source Code), and has been released under the auspice of the GNU Lesser General Public License (LGPL).

**Figure 1:**
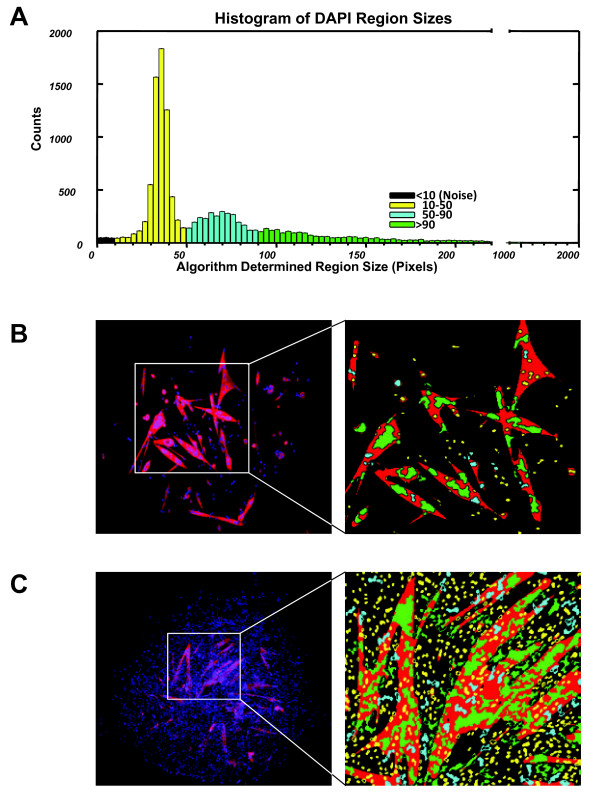
**Flow chart of program algorithm. (A)** G-Tool algorithm flowchart. **(B)** Left: An example satellite cell colony. Right: G-Tool rasterized version of the colony. Red denotes myosin heavy chain (MHC) + cytoplasm. Nuclei are blue. **Flow chart of program algorithm. (A)** G-Tool algorithm flowchart. **(B)** Left: An example satellite cell colony. Right: G-Tool rasterized version of the colony. Red denotes myosin heavy chain (MHC) + cytoplasm. Nuclei are blue.

The function of the algorithm can be divided into three elements: the first handles nuclear counting, the second handles the identification of sarcomeric MHC + regions; and the third combines the results of the first two and identifies the number of MHC + myofibers that are mononucleated, binucleated, or multinucleated (defined as having more than two nuclei in one fiber). G-Tool uses features the MATLAB Image Processing Toolbar (IPT) for these computations. A flowchart illustrating the algorithm is shown in Figure 1A. A comparison between a typical satellite cell image and the rasterized output from the GUI is shown in Figure 1B.

### Identification and quantification of nuclei

**Figure 2:**
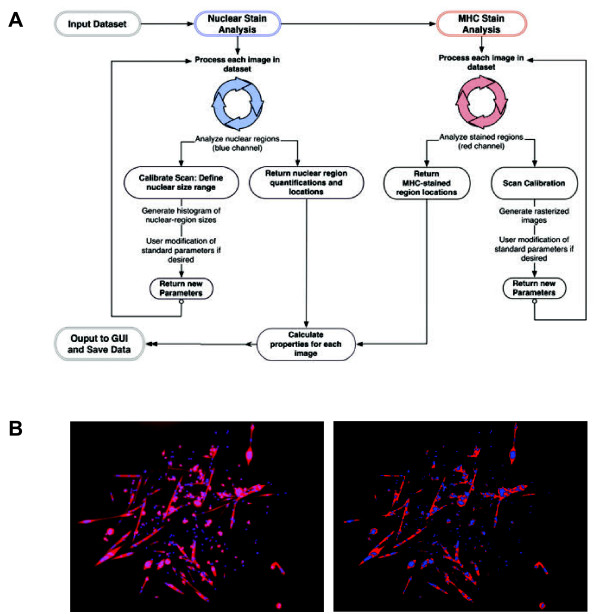
**4',6-Diamidino-2-phenylindole (DAPI) region sizes interpreted by G-Tool. (A)** Histogram showing DAPI + region sizes identified by G-Tool in a dataset containing >23,000 nuclei (C57BL/6 hindlimb satellite cells). The histogram is color coded to reflect the different sizes of nuclei that are visible in the library of images. **(B)** Left: An example satellite cell colony. Right: G-Tool rasterized version (magnified) of this relatively simple colony. Red denotes myosin heavy chain (MHC) + cytoplasmic regions. The DAPI + regions are colored according to the scheme defined by the histogram in (A). **(C)** Left: An example of a very dense satellite cell colony. Right: G-Tool rasterized version (magnified). Human counting of the regions of densely packed nuclei would be highly variable.

All of the CFC parameters are based on nuclear number. In mapping DAPI + areas to identify nuclei, it was apparent that the size of each DAPI + area could vary widely. In order to identify bona fide single nuclei, G-Tool analyzes the size distribution of all nuclei within the set of images. A typical size distribution is shown in Figure 2A. Within this distribution, the majority of nuclei fall within a narrow peak at small size, however a significant number of larger-sized DAPI + domains are present, typically as a second broader peak followed by a long tail representing relatively few but very large DAPI + domains. By visual inspection of nuclei within the distribution, it was obvious that most DAPI + areas within the second broad peak represent what the human eye might identify as doublets or triplets, while larger DAPI + areas represent larger numbers of overlapping nuclei that the human eye would have difficulty assigning a value to (Figure 2B). This is in fact a significant source of variability associated with human counting of CFC images. To assign a nuclear quantity to each DAPI + area, the assumption is made that DAPI + areas within the first peak are single nuclei, and a statistical probability size is assigned to nuclei outside of this peak based on how large each is compared to the mean size of DAPI + areas found within the first peak. To determine whether a nucleus is within or outside of an MHC + area (also sometimes difficult and variable with human counting, especially in dense colonies where myotubes may be adjacent to many single cells), the geometric center of each DAPI + area is identified, and if this center falls within an MHC + area, the entire DAPI + area is considered to be within an MHC + cytoplasm.

### Comparison to human counting

To gauge the ability of the algorithm to act as a human surrogate for analyzing a large number of plates and colonies we compared human with G-Tool counting over a large number of colony images. We determined the following properties from each image: the number of nuclei present on the image, the number of cells that stained negative for MHC, and the number of MHC + cells with one, two, and three or more nuclei. Using these properties we defined the coefficient of differentiation, D_f_, as the frequency of nuclei within a MHC + cytoplasm. This metric represents the state of differentiation of a satellite cell colony on a scale from zero to one (Equation 1A). The fusion index, U_i_, represents the frequency of nuclei in the colony that have definitively gone through a fusion event (cells with greater than two nuclei are considered to be definitive fusion products as cells with two nuclei could be dividing), and is also on a scale of zero to one (Equation 1B). U_i_ acts as a second order indicator of colony differentiation, as fusion depends on both the state of differentiation and cell density of the colony.

(1) $$D_{f}=1-\frac{\#Stain\;Negative}{Total\;Nuclei’}\;U_{i}=\frac{\sum Nuclei\;within\;cells\;of>2\;nuclei}{Total\;Nuclei’}$$

**Figure 3:**
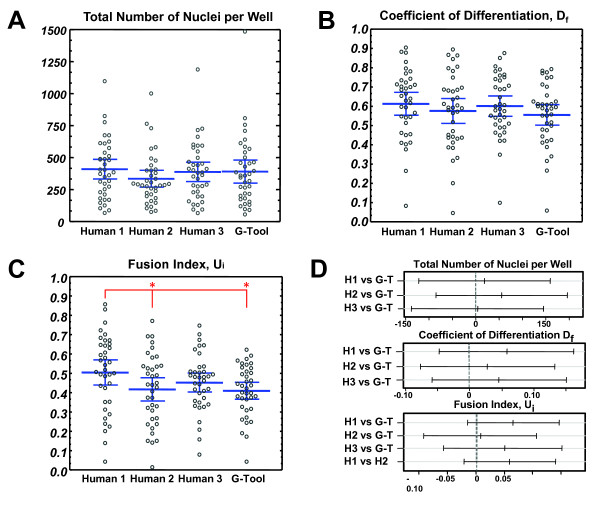
**Manual vs G-Tool counting. (A)** Number of nuclei per colony, counted manually by three different individuals, and compared to G-Tool. Blue bars represent mean and 95% confidence intervals. **(B)** Coefficient of differentiation, D_f_, counted as in (A). Blue bars represent mean and 95% confidence intervals. **(C)** Fusion index, U_i_, counted as in (A). Blue bars represent mean and 95% confidence intervals. Pairwise paired *t* test results: human 1 vs human 2 (*P* value = 4E-4)*, human 1 vs human 3 (*P* value = 0.22), human 1 vs G-Tool (*P* value = 6E-4)*, human 2 vs human 3 (*P* value = 0.40), human 2 vs G-Tool (*P* value = 0.77), human 3 vs G-Tool (*P* value = 0.22). *Paired *t* test shows a statistically significant difference between means at 95% confidence interval. **(D)** Statistical analysis: the differences between G-Tool and humans 1 to 3 is not statistically significant for eight of nine parameters. Tukey pairwise comparison test is shown comparing G-Tool counting to human counting for the total number of nuclei per well, the coefficient of differentiation, and the fusion index. If the pairwise range contains zero, the difference between the pair is not statistically significant. Blue bars represent mean and 95% confidence intervals. By Shapiro-Wilke test, data for D_f_ and U_i_ were found to be normally distributed while number of nuclei showed a slight deviation from normality. We performed analysis of variance (ANOVA) and Tukey honestly significant difference (HSD) test on square root-transformed number of nuclei data and found that the same results were obtained with this normalized data set.

We performed CFC assays using satellite cells from total hindlimb digests of C57BL/6 congenic Pax7-ZsGreen mice. A total of 37 colonies were imaged and evaluated manually by 3 different individuals and G-Tool. As expected, data sets generated by each individual were similar but not identical (Figure 3). This is because many aspects of these images are highly subjective. For example, although multinucleated myotubes stain strongly for sarcomeric myosin (MHC), mononuclear cells range from completely negative to strongly positive, and the distinction between stained and unstained is arbitrary. In dense colonies, mononuclear cells may be adjacent to myotubes, making it difficult to know whether their nuclei belong to the myotube or not. In all but one pairwise comparison (Figure 3D), G-Tool results were not statistically different from human counting. In this case, although the Fusion Index data were found to be different between human 1 and G-Tool, they were also found to be statically significantly different between human 1 and human 2. This variability between human counts only underscores the need for repeatable, consistent measuring. Because many aspects of image analysis are arbitrary, it can be difficult to assert what the ‘correct’ value should be. A key advantage of using an algorithm to determine CFC metrics is that the algorithm is consistent. By not varying over time and by giving identical results if images are reanalyzed, one element of variance inherent in CFC assays is eliminated. However, the components of variability due to experimental variation (efficiency of immunostaining, quality of microscope, and so on) remain. To deal with this aspect, G-Tool allows the user to adjust sensitivities, however care must be taken to ensure that the output after modifying various algorithm parameters is reasonable, for example by examining the analysis of a simple colony with few overlapping features.

### CFC assay in reduced vs ambient O_2_

We applied the G-Tool algorithm to realize a CFC experiment in which we compared colony-forming potential at low oxygen concentrations to colony growth at ambient oxygen concentrations. Six plates were cultured under low oxygen conditions using a hypoxic chamber filled with a low oxygen gas mixture (3% O_2_, 5% CO_2_, 92% N_2_) and six plates were cultured at 37°C in ambient oxygen conditions (21% O_2_, 5% CO_2_).

**Figure 4:**
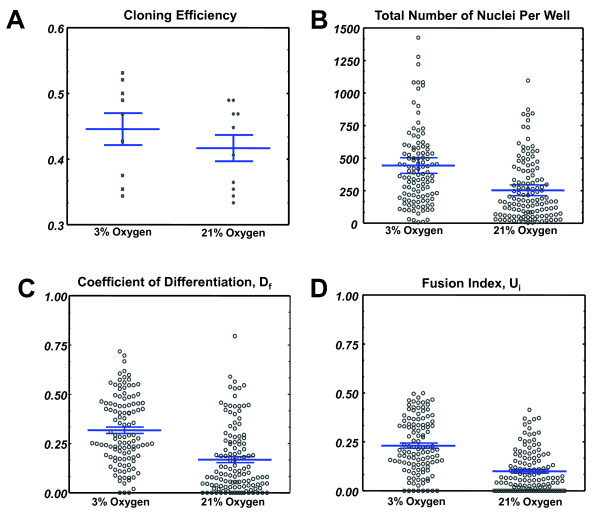
**Colony-forming cells (CFCs) assayed at 3% vs 21% O**_**2**_**. (A)** Cloning efficiency (frequency of single satellite cells that generated a colony in a single well) under low (3%) oxygen and ambient (20%) oxygen. Blue bars represent mean and 95% confidence intervals. **(B)** Number of nuclei per colony for the low and ambient oxygen groups. Blue bars represent mean and 95% confidence intervals. **(C)** The coefficient of differentiation, D_f_, for the two experimental groups. Blue bars represent mean and 95% confidence intervals. **(D)** The fusion index, U_i_, for the two experimental groups. Blue bars represent mean and 95% confidence interval. Blue bars represent mean and 95% confidence intervals.

The cloning efficiency of satellite cells (frequency of wells in which a colony was present) was comparable in the normal (21%) oxygen vs reduced (3%) oxygen group (0.42 vs 0.38, respectively). After processing the images, we found that the number of cells per colony was greater in the low oxygen group (Figure 4A, *P* = 0.000004). In addition, we discovered a remarkable increase in both the frequency of nuclei within an MHC + cytoplasm (D_f_) and the fusion index (U_i_) in colonies grown in low oxygen, shown in Figure 4B,C (*P* = 6.2 × 10^-10^ and 1.9 × 10^-7^, respectively) This indicates that although there is more proliferation occurring under low oxygen, there is also both quantitatively and proportionally more differentiation taking place.

### CFCs from the diaphragm are more proliferative and show less differentiation

**Figure 5:**
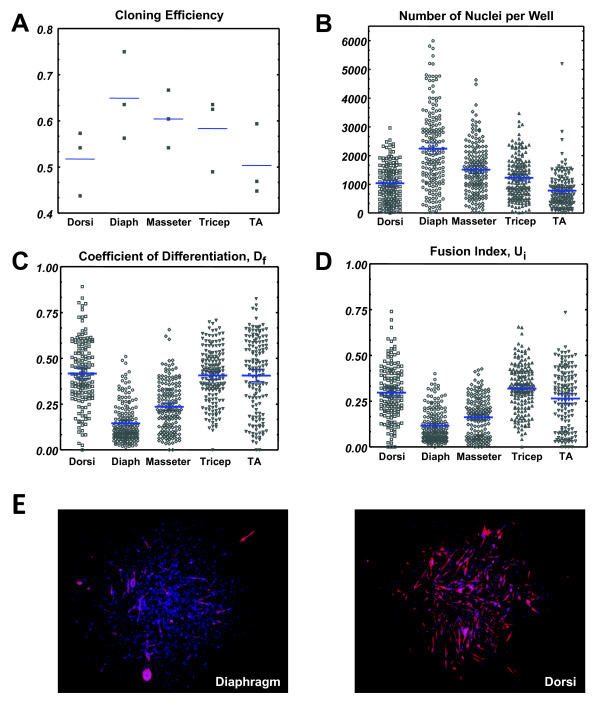
**Colony-forming cell (CFC) analysis of different muscle groups. (A)** Cloning efficiency for all five muscle groups across three replicates (independent mice). Cloning efficiency represents the percentage of wells in a 96-well plate that show satellite cell colony formation. Each data point represents one plate. Blue bars represent the mean. **(B)** The total number of nuclei per colony for each of five muscle groups. Blue bars represent mean and 95% confidence intervals. **(C)** The coefficient of differentiation, D_f_ for all five muscle groups. Blue bars represent mean and 95% confidence intervals. **(D)** The fusion index, U_i_ for all five muscle groups. Blue bars represent mean and 95% confidence intervals. **(E)** Example colonies. Left: typical diaphragm colony. Right: typical dorsi colony showing high differentiation percentage. Blue bars represent mean and 95% confidence intervals. Diaph = diaphragm; TA = tibialis anteriors.

We wished to determine whether satellite cells from different muscles were equivalent in myogenic potential, or had intrinsic differences that could be revealed by the CFC assay. We therefore harvested individual muscles of different anatomical locations from 1-month-old mice, including masseter, diaphragm, latissimus dorsi, tricep, and tibialis anterior muscles. Cloning efficiencies were comparable between muscle groups at approximately 60%, but diaphragm consistently showed the highest cloning efficiency (Figure 5A). The CFC analysis evaluated 823 colonies comprising over 300,000 nuclei in total. Remarkably, all CFC parameters, average number of nuclei, D_f_ and U_i_ varied widely among satellite cells of different muscle groups (Figure 5B-E). In particular, the colonies derived from diaphragm satellite cells were distinct. They were on average much larger (*P* = 1.6 × 10^-9^ comparing to masseter, the next largest) and showed significantly less differentiation, both in terms of nuclei within MHC + cytoplasm, (D_f_; *P* <1 × 10^-10^, compared to masseter) as well as fusion index (U_i_; *P* = 1.1 × 10^-6^, compared to masseter). With regard to D_f_ and U_i_, the masseter colonies showed lower levels of differentiation compared to the dorsi, tricep and TA muscles. These data suggest that the satellite cells of different muscle groups have distinct anabolic potential, with those of the diaphragm being capable of producing more muscle tissue on a per cell basis, that is, having the greatest regenerative capacity. We speculate below on why this might be the case.

## Discussion

By enabling the assignment of metrics of proliferation (nuclear number) and differentiation (MHC + nuclei, D_f_; and fusion index; U_i_) to large numbers of satellite cell-derived colonies, G-Tool enables CFC analysis to be applied rigorously to skeletal muscle. Unlike hematopoietic CFCs, muscle-derived CFCs are unilineage, however heterogeneity within the satellite cell compartment manifests as CFCs having greater or lesser proliferative potential and higher or lower rates of differentiation. While CFC analysis is not new to the muscle field [12], its application is most easily applied to screening presence or absence of colonies, for example within fractions isolated by FACS [13]. In cases where effects on colony composition have been investigated, screening individual cells from large numbers of colonies is technically limiting, therefore gross measures of differentiation have been estimated [14], or limited numbers of colonies evaluated [15]. Through automated counting of nuclear number and identifying cells expressing sarcomeric myosin, G-Tool allows quantitative comparisons between colonies, which facilitates the study of heterogeneity within the satellite cell compartment. While some heterogeneity must be due to stochastic mechanisms (equivalent cells expressing different fates due to intrinsic randomness), there is strong evidence for intrinsic heterogeneity within the satellite cell compartment. While many, perhaps most, satellite cells express Myf5 [16], a significant subpopulation exists that has never expressed Myf5, and when transplanted, these contribute more potently to the satellite cell compartment, and can generate Myf5+ progeny [17]. Similar functional heterogeneity has been demonstrated in subsets of Pax7+ satellite cells expressing ABCG2 and Sca-1 [18]. Accordingly, when CFCs are plated and allowed to grow into differentiated colonies, a spectrum of colony sizes and differentiation levels are seen.

In these CFC assays, we did not induce differentiation, rather it occurred spontaneously. This spontaneous differentiation distinguishes these primary cell cultures from those of myoblast cell lines, for example, C2C12 or MM14 cells or even conventional presenescent cultures of primary myoblasts, which are artificially locked into a proliferative state, and exit from this state is only achieved by subjecting cells to the extreme stress of growth factor withdrawal. Therefore, factors identified through the CFC assay that impact differentiation or proliferation are far more likely to be physiologically relevant. We focused on one external variable: oxygen concentration. We found that colonies were much smaller when cultured in ambient 21% O_2_, than when cultured in low (3%) O_2_, but they were also much less differentiated. The increase in proliferation when oxygen levels were kept low was not unexpected [19,20]. However the increased rate of terminal myogenic differentiation was not predicted. Because in immortalized myoblast systems, differentiation is essentially induced by stress, excessive differentiation is commonly interpreted as a response to suboptimal culture conditions. That we observe more differentiation at 3% O_2_ means either that 3% O_2_ is suboptimal compared to 21% O_2_, or that this interpretation is not valid for primary cells in the initial stages of culture, that is, rapid differentiation of at least a subpopulation of transit-amplifying progeny is a normal response. Given the wealth of information regarding the impact of oxygen levels in culture of primary cells [21,22], the fact that 21% O_2_ is demonstrably non-physiological [23], and indeed that cell proliferation was negatively affected by 21% O_2_ in our study, we favor the latter explanation. In conventional primary myoblast culture, spontaneous differentiation is indeed seen at the initial plating, but is lost as the culture is expanded due to selection for a subpopulation of cells that is locked into a non-physiological non-differentiating, proliferative state. Because CFC analysis focuses on a time point a few days after plating, this culture artifact is largely avoided. This is a major strength of the CFC approach.

As different muscles have different regenerative requirements, whether specific differences are programmed into the satellite cells of different muscles is an important question. A recent study compared masseter to EDL and found micromanipulated cells derived from isolated myofibers to have a wide range of colony sizes and frequencies of Pax7+/MyoD + progeny, with greater variability in the masseter [15]. While it is clear that the satellite cells of different muscle groups have different developmental histories, with the myogenic program being initiated by different upstream regulators [24,25], it is not clear whether this imbues them with cell-autonomous functional differences, or whether regenerative differences that may exist are programmed by the environment. Strong support for the latter idea comes from heterotopic transplantations, in which satellite cells of the extraocular muscles produced differentiated muscle characteristic of the TA when transplanted into the TA muscle [25]. We performed clonal assays from five different muscle groups and found that each had a different average size, and differentiation rate. Within this group, the diaphragm was remarkable in terms of the large size of clones and the relatively lower rates of differentiation within each clone. Across the five muscle groups, there was an inverse relationship between the average size and differentiation rate, which is consistent with the idea that propensity to differentiate plays a role in specifying clone size: as differentiation is associated with withdrawal from the cell cycle, greater differentiation leaves fewer cells capable of expanding the progenitor pool. Assuming behavior *in vitro* reflects regenerative potential *in vivo*, a diaphragm satellite cell, whose transit amplifying progeny are capable of expanding into a very large clone before differentiating, would have greater regenerative potential than a satellite cell of the TA.

What is responsible for these differences is not clear, however, diaphragm satellite cells are distinguished from those of most other muscles in that they express Pax3 when resting [4] and they also express higher levels of Pax3 when cultured [10]. It would be interesting to evaluate whether elimination or reduction of Pax3 would alter the diaphragm CFCs such that they became more like those of other muscle groups. The diaphragm is a rather unique muscle in terms of regeneration. It never rests, therefore regeneration after injury is intrinsically more difficult here than in any other muscle of the body. In addition, it is extremely plastic; atrophy ensues after only a short time of disuse, as when on mechanical ventilation [26], but recovery after such atrophy can be robust. These unique features may require satellite cells of greater regenerative potential.

Since cloning efficiency is less than 100%, meaning that many cells do not form colonies, it is important to acknowledge the caveat that conclusions obtained from CFC analysis are subject to the assumption that sampling (in this case formation of colonies) is random. Care should be taken to consider what influences may bias sampling, and therefore whether this is a reasonable assumption. This problem is not unique to CFC analysis, but shared with any study that makes conclusions based on cells assayed retrospectively, for example transplantation studies. In this study, we note that cloning efficiency was highest for those muscle groups that gave the largest, least differentiated colonies. A caveat to keep in mind is that a systematic difference in treatment, for example differential exposure to disaggregating enzymes due to the different sizes/shapes of different muscle groups, could affect the propensity of cells to form colonies or alter their proliferative/differentiation potential.

## Conclusions

In summary, G-Tool allows CFC analysis to be accomplished rapidly and efficiently. This facilitates both evaluation of *ex vivo* culture conditions as we have shown with O_2_ levels, as well as evaluation of intrinsic differences between types of satellite cells, as shown for different muscle groups. The latter experiment in particular, where several hundred thousand nuclei were evaluated, would have taken several weeks of full-time human counting to complete. In addition to the time saved, an automated CFC assay avoids the variability associated with human image interpretation. G-Tool is open source, released under the auspice of the GNU Lesser General Public License, and therefore may be further enhanced by interested members of the community. Valuable enhancements might include improvements to accuracy of measuring nuclear count (possibly based on evaluating shape of DAPI + areas in addition to size) or incorporation of heuristic methods to defining MHC + areas. Metrics based on dual fluorescent channels, for example measuring the presence of a nuclear factor such as Myf5 or myogenin, might provide additional discriminatory power to CFC analysis.

## Competing interests

The authors declare they have no competing interests.

## Authors’ contributions

JI wrote G-Tool, designed and conducted experiments and contributed to writing the manuscript. RA and KH conducted experiments and analyzed data. JZ analyzed the data and contributed to writing the manuscript. MK supervised the overall project, designed experiments, analyzed the data and wrote the paper. All authors read and approved the final manuscript.

## Supplementary Material

Additional file 1 **G-Tool Source Code.** Java and MATLAB Source Codes are included. Click here for file
