# Supplementary material for: Satellite cell heterogeneity revealed by G-Tool, an open algorithm to quantify myogenesis through colony-forming assays
Source: Skelet Muscle. 2012 Jun 15;2:13. doi: 10.1186/2044-5040-2-13 (PMC3439689; doi:10.1186/2044-5040-2-13)
Supplement: Additional file 1 — G-Tool Source Code. Java and MATLAB Source Codes are included. [file 2044-5040-2-13-S1.zip › G-Tool Sourcecode and PDF files/PDF files of code/JAVA - GUI/GTOOL.pdf]

```
/*%     This file is part of GTOOL. AUTHOR: JOSEPH IPPOLITO, THE UNIVERSITY
%       OF MINNESOTA. GTOOL is free software: you can redistribute it
%   and/or modify
%       it under the terms of the GNU General Public License as published
%   by the Free Software Foundation, either version 3 of the License, or
%       (at your option) any later version.
%       GTOOL is distributed in the hope that it will be useful,
%   but WITHOUT ANY WARRANTY; without even the implied warranty of
%   MERCHANTABILITY or FITNESS FOR A PARTICULAR PURPOSE. SEE THE GNU
%   GENERAL PUBLIC LISCENCE FOR MORE DETAILS.
%       You should have received a copy of the GNU General Public License
%   along with GTOOL. If not see see <http://www.gnu.org/licenses/>. */
package gtool;
import java.beans.PropertyVetoException;
import java.util.logging.Level;
import java.util.logging.Logger;
import javax.swing.JInternalFrame;
import javax.swing.JDesktopPane;
import javax.swing.JFrame;
import java.awt.*;
public class GTOOL extends JFrame{
    JDesktopPane desktop;
    private JInternalFrame mainframe1;
    private Menubar menubar = new Menubar();
    public GTOOL() {
        super("GTOOL");
        //Make the big window be indented 50 pixels from each edge
        //of the screen.
       // System.setProperty("apple.laf.useScreenMenuBar", "true");
        mainframe1 = new JInternalFrame("", false, false,  false, false);
        ((javax.swing.plaf.basic.BasicInternalFrameUI) mainframe1.getUI
()).setNorthPane(null);


        int inset = 50;
        Dimension screenSize = Toolkit.getDefaultToolkit().getScreenSize();
        setBounds(inset, inset,screenSize.width  -
inset*2,screenSize.height - inset*2);
        //Set up the GUI.

        //CREATE THE VIRTUAL DESKTOP ENVIRONMENT
        desktop = new JDesktopPane(); //a specialized layered pane
        setLayout(new FlowLayout());
        //createFrame(); //create first "window"
        setContentPane(desktop);
        setJMenuBar(menubar.createMenuBar2());
```

```
        desktop.setDragMode(JDesktopPane.OUTLINE_DRAG_MODE);
        //CREATE THE INTERNAL FRAME THAT HOLDS THE PROGRAM

        mainframe1.putClientProperty("JInternalFrame.isPalette",
Boolean.TRUE);
        mainframe1.setBounds(350, 150, 100, 100);
        mainframe1.getContentPane().add(new MainProgram
(),BorderLayout.CENTER);
        mainframe1.setVisible(true);
        desktop.add(mainframe1);
        mainframe1.setVisible(true);
            try {
            mainframe1.setMaximum(true);
        } catch (PropertyVetoException ex) {
            Logger.getLogger(GTOOL.class.getName()).log(Level.SEVERE, null,
ex);
        }

    }



    private static void createAndShowGUI() {
        //Make sure we have nice window decorations.
        JFrame.setDefaultLookAndFeelDecorated(false);
        //Create and set up the window.
        GTOOL frame = new GTOOL();
        frame.setDefaultCloseOperation(JFrame.EXIT_ON_CLOSE);
        frame.setVisible(true);
    }

    public static void main(String[] args) {
        //Schedule a job for the event-dispatching thread:
        //creating and showing this application's GUI.
        javax.swing.SwingUtilities.invokeLater(new Runnable() {
            public void run() {
                createAndShowGUI();
            }
        });
    }



}
```
