# Supplementary material for: Satellite cell heterogeneity revealed by G-Tool, an open algorithm to quantify myogenesis through colony-forming assays
Source: Skelet Muscle. 2012 Jun 15;2:13. doi: 10.1186/2044-5040-2-13 (PMC3439689; doi:10.1186/2044-5040-2-13)
Supplement: Additional file 1 — G-Tool Source Code. Java and MATLAB Source Codes are included. [file 2044-5040-2-13-S1.zip › G-Tool Sourcecode and PDF files/PDF files of code/JAVA - GUI/Image_Selection_Panel.pdf]

```
/*%     This file is part of GTOOL. AUTHOR: JOSEPH IPPOLITO, THE UNIVERSITY
%       OF MINNESOTA. GTOOL is free software: you can redistribute it
%   and/or modify
%       it under the terms of the GNU General Public License as published
%   by the Free Software Foundation, either version 3 of the License, or
%       (at your option) any later version.
%       GTOOL is distributed in the hope that it will be useful,
%   but WITHOUT ANY WARRANTY; without even the implied warranty of
%   MERCHANTABILITY or FITNESS FOR A PARTICULAR PURPOSE. SEE THE GNU
%   GENERAL PUBLIC LISCENCE FOR MORE DETAILS.
%       You should have received a copy of the GNU General Public License
%   along with GTOOL. If not see see <http://www.gnu.org/licenses/>. */
package gtool;
import javax.swing.event.*;
import java.awt.event.*;
import java.io.*;
import java.awt.*;
import java.util.*;
import javax.swing.*;

class SortedListModel extends AbstractListModel {
  SortedSet<Object> model;

  public SortedListModel() {
    model = new TreeSet<Object>();
  }

    @Override
  public int getSize() {
    return model.size();
  }

    @Override
  public Object getElementAt(int index) {
    return model.toArray()[index];
  }

  public void add(Object element) {
    if (model.add(element)) {
      fireContentsChanged(this, 0, getSize());
    }
  }

  public void addAll(Object elements[]) {
    Collection<Object> c = Arrays.asList(elements);
    model.addAll(c);
```

```
    fireContentsChanged(this, 0, getSize());
  }

  public void clear() {
    model.clear();
    fireContentsChanged(this, 0, getSize());
  }

  public boolean contains(Object element) {
    return model.contains(element);
  }

  public Object firstElement() {
    return model.first();
  }

  public Iterator iterator() {
    return model.iterator();
  }

  public Object lastElement() {
    return model.last();
  }

  public boolean removeElement(Object element) {
    boolean removed = model.remove(element);
    if (removed) {
      fireContentsChanged(this, 0, getSize());
    }
    return removed;
  }
}

class Image_Selection_Panel extends JPanel {

  static int openFrameCount = 0;
  static final int xOffset = 30, yOffset = 30;

  private JList sourceList;

  private SortedListModel sourceListModel;

  private JList destList;

  private SortedListModel destListModel;
```

```
  private JButton addButton;

  private JButton removeButton;

  private JPanel imagepaneloutput_panel = new JPanel();

  private JLabel imagepaneloutput_label = new JLabel("hello????");

  private JButton processButton;
  private JButton calibrateButton;

  public File dir;

  public JPanel image_panel;
  private JLabel output_label1;
  private JPanel test_output_panel;
  private JPanel intermediate = new JPanel();

  private Image image;
  private Icon icon;

  private JLabel available_images = new JLabel("Available Images: No
Images");
  private JLabel selected_images  = new JLabel("Selected Images: No
Images");

  private Menubar menubar = new Menubar();
  private Input_Settings_Panel Input_Settings_Panel1 = new
Input_Settings_Panel();
  private JButton run_calibration    = new JButton("Calibrate..");

  public Image_Selection_Panel() {
   this.setLayout(new GridLayout(0, 2));
   sourceListModel = new SortedListModel();
   sourceList = new JList(sourceListModel);
   addButton = new JButton(">>");
   addButton.addActionListener(new AddListener());
   removeButton = new JButton("<<");
   removeButton.addActionListener(new RemoveListener());
   destListModel = new SortedListModel();
   destList = new JList(destListModel);

   destList.addListSelectionListener(new DestListSelectionHandler());

    MouseListener mouseListener = new MouseAdapter() {
            @Override
```

```
  public void mouseClicked(MouseEvent e) {
      if (e.getClickCount() == 2) {
          int index = destList.locationToIndex(e.getPoint());
          removeButton.doClick();
       }
  }
  };

 destList.addMouseListener(mouseListener);

  MouseListener mouseListener2 = new MouseAdapter() {
          @Override
  public void mouseClicked(MouseEvent e) {
      if (e.getClickCount() == 2) {
          int index = sourceList.locationToIndex(e.getPoint());
          addButton.doClick();
       }
  }
  };
 sourceList.addMouseListener(mouseListener2);


 JPanel leftPanel = new JPanel(new BorderLayout());
 leftPanel.setPreferredSize(new Dimension(300, 300));

 leftPanel.add(available_images, BorderLayout.NORTH);
 leftPanel.add(new JScrollPane(sourceList), BorderLayout.CENTER);
 leftPanel.add(addButton, BorderLayout.SOUTH);


  JPanel rightPanel = new JPanel(new BorderLayout());
  rightPanel.setPreferredSize(new Dimension(300, 300));

  rightPanel.add(selected_images, BorderLayout.NORTH);
  rightPanel.add(new JScrollPane(destList), BorderLayout.CENTER);
  rightPanel.add(removeButton, BorderLayout.SOUTH);
  this.add(leftPanel,BorderLayout.WEST);
  this.add(rightPanel,BorderLayout.EAST);
}

    public JLabel getavailableLabel()
{
   return available_images;
}

    public JLabel getselectedLabel()
```

```
    {
        return selected_images;
    }




    public void clearSourceListModel() {
      sourceListModel.clear();
    }

    public void clearDestinationListModel() {
      destListModel.clear();
    }

    public void addSourceElements(ListModel newValue) {
      fillListModel(sourceListModel, newValue);
    }

    public void setSourceElements(ListModel newValue) {
      clearSourceListModel();
      addSourceElements(newValue);
    }

    public void addDestinationElements(ListModel newValue) {
      fillListModel(destListModel, newValue);
    }

    private void fillListModel(SortedListModel model, ListModel newValues) {
      int size = newValues.getSize();
      for (int i = 0; i < size; i++) {
        model.add(newValues.getElementAt(i));
      }
    }

    public void addSourceElements(Object newValue[]) {
      fillListModel(sourceListModel, newValue);
            available_images.setText("Available Images: " +
  sourceList.getModel().getSize());

    }

    public void setSourceElements(Object newValue[]) {
      clearSourceListModel();
      addSourceElements(newValue);
    }
```

```
  public void addDestinationElements(Object newValue[]) {
    fillListModel(destListModel, newValue);
  }

  private void fillListModel(SortedListModel model, Object newValues[]) {
    model.addAll(newValues);
  }

  private void clearSourceSelected() {
    Object selected[] = sourceList.getSelectedValues();
    for (int i = selected.length - 1; i >= 0; --i) {
      sourceListModel.removeElement(selected[i]);
    }
    sourceList.getSelectionModel().clearSelection();
  }

  private void clearDestinationSelected() {
    Object selected[] = destList.getSelectedValues();
    for (int i = selected.length - 1; i >= 0; --i) {
      destListModel.removeElement(selected[i]);
    }
    destList.getSelectionModel().clearSelection();
  }


  private class AddListener implements ActionListener {
        @Override
    public void actionPerformed(ActionEvent e) {
      Object selected[] = sourceList.getSelectedValues();
      addDestinationElements(selected);
      clearSourceSelected();

      available_images.setText("Available Images: " + sourceList.getModel
().getSize());
      selected_images.setText("Selected Images: " + destList.getModel
().getSize());


      if(destList.getModel().getSize() != 0){
          processButton.setEnabled(true);
          calibrateButton.setEnabled(true);
          menubar.placedir(processButton);
          Input_Settings_Panel1.placedir(processButton,
calibrateButton,destList);
          destList.setSelectedIndex(0);
```

```
      }

    }
  }

  private class RemoveListener implements ActionListener {
        @Override
    public void actionPerformed(ActionEvent e) {
      Object selected[] = destList.getSelectedValues();
      addSourceElements(selected);
      clearDestinationSelected();

      available_images.setText("Available Images: " + sourceList.getModel
().getSize());
      selected_images.setText("Selected Images: " + destList.getModel
().getSize());
      run_calibration = Input_Settings_Panel1.setCalibrationButton();

      if(destList.getModel().getSize() == 0){
          processButton.setEnabled(false);
          calibrateButton.setEnabled(false);

      }else{
          destList.setSelectedIndex(0);
      }


    }
  }


    private class DestListSelectionHandler implements ListSelectionListener
{
        @Override
     public void valueChanged(ListSelectionEvent e) {
            Object selected[] = destList.getSelectedValues();
            int lengthofpopulatedlist = selected.length;
            if(lengthofpopulatedlist ==1){
                 String image_path = dir.toString() + "\\"+ selected[0];
                 //just one selection
                 System.out.println("image selected: " + selected[0]);
                 System.out.println("Image path: " + image_path);
                  imageOutputPanelCreator output_image = new
imageOutputPanelCreator(image_path);
                  intermediate.removeAll();
                  intermediate.add(output_image);
```

```
                    image_panel.add(intermediate,BorderLayout.CENTER);

                    image_panel.setVisible(true);
                    image_panel.revalidate();
                    output_label1.setIcon(output_image.getIcon());
              }else{
                   output_label1.setText("Multiple Images Selected");
              }
  }
}

   @Override
    public void paintComponent(Graphics g) {
        super.paintComponent(g);
        int w = getWidth();     // get width of panel.
        int h = getHeight();
        g.drawImage(image,25,15,w-45,h-45,null);
    }

    public JPanel outputLabelwindow()
   {

       imagepaneloutput_panel.add(imagepaneloutput_label);
       return imagepaneloutput_panel;

   }


    public void placedir(File directory, JPanel panel, JLabel label, JPanel
panel2,JButton button, JButton button2){
        test_output_panel = panel2;
        output_label1 = label;
        image_panel = panel;
        dir = directory;
        processButton = button;
        calibrateButton = button2;
    }

    public JList getDestList(){
        return destList;
    }

    public JButton getprocessButton(){
        return processButton;
    }
```

```
}
```
