# Supplementary material for: Satellite cell heterogeneity revealed by G-Tool, an open algorithm to quantify myogenesis through colony-forming assays
Source: Skelet Muscle. 2012 Jun 15;2:13. doi: 10.1186/2044-5040-2-13 (PMC3439689; doi:10.1186/2044-5040-2-13)
Supplement: Additional file 1 — G-Tool Source Code. Java and MATLAB Source Codes are included. [file 2044-5040-2-13-S1.zip › G-Tool Sourcecode and PDF files/PDF files of code/JAVA - GUI/imageOutputPanelCreator.pdf]

```java
//%     This file is part of GTOOL. AUTHOR: JOSEPH IPPOLITO, THE UNIVERSITY
//%     OF MINNESOTA.
//%
//%     GTOOL is free software: you can redistribute it and/or modify
//%     it under the terms of the GNU General Public License as published
by
//%     the Free Software Foundation, either version 3 of the License, or
//%     (at your option) any later version.
//%
//%     GTOOL is distributed in the hope that it will be useful,
//%     but WITHOUT ANY WARRANTY; without even the implied warranty of
//%     MERCHANTABILITY or FITNESS FOR A PARTICULAR PURPOSE.  See the
//%     GNU General Public License for more details.
//%
//%     You should have received a copy of the GNU General Public License
//%     along with GTOOL.  If not, see <http://www.gnu.org/licenses/>.
package gtool;
import java.awt.*;
import java.awt.image.*;
import java.io.*;
import javax.imageio.*;
import javax.swing.*;

public class imageOutputPanelCreator extends JLabel {
   private BufferedImage image = null;
   private Icon icon = null;

    public imageOutputPanelCreator(String path) {

      //  String tpath = "/Users/Joseph/Pictures/IMG_0004 1.JPG";

        //setBorder(BorderFactory.createLineBorder(Color.black));
        if(path!=null){

        try {
        image = ImageIO.read(new File(path));

        } catch (IOException ioe) {
        System.out.println(ioe);
        System.exit(0);
        }
        icon = new ImageIcon(image);
    }


    }
```

```
    @Override
    public Dimension getPreferredSize() {
        return new Dimension(500,500);
    }

    @Override
    public void paintComponent(Graphics g) {
        super.paintComponent(g);
        int w = getWidth();     // get width of panel.
        int h = getHeight();
        g.drawImage(image,25,15,w-45,h-45,null);

    }
}
```
