# Supplementary material for: Satellite cell heterogeneity revealed by G-Tool, an open algorithm to quantify myogenesis through colony-forming assays
Source: Skelet Muscle. 2012 Jun 15;2:13. doi: 10.1186/2044-5040-2-13 (PMC3439689; doi:10.1186/2044-5040-2-13)
Supplement: Additional file 1 — G-Tool Source Code. Java and MATLAB Source Codes are included. [file 2044-5040-2-13-S1.zip › G-Tool Sourcecode and PDF files/PDF files of code/JAVA - GUI/Input_Settings_Panel.pdf]

```
/*%     This file is part of GTOOL. AUTHOR: JOSEPH IPPOLITO, THE UNIVERSITY
%       OF MINNESOTA. GTOOL is free software: you can redistribute it
%   and/or modify
%       it under the terms of the GNU General Public License as published
%   by the Free Software Foundation, either version 3 of the License, or
%       (at your option) any later version.
%       GTOOL is distributed in the hope that it will be useful,
%   but WITHOUT ANY WARRANTY; without even the implied warranty of
%   MERCHANTABILITY or FITNESS FOR A PARTICULAR PURPOSE. SEE THE GNU
%   GENERAL PUBLIC LISCENCE FOR MORE DETAILS.
%       You should have received a copy of the GNU General Public License
%   along with GTOOL. If not see see <http://www.gnu.org/licenses/>. */
package gtool;
import java.awt.*;
import javax.swing.event.*;
import javax.swing.*;
import java.awt.Color;
import java.awt.event.ActionEvent;
import java.awt.event.ActionListener;
import javax.swing.border.Border;

public class Input_Settings_Panel extends JPanel {

    static public JButton processButton;
    static public JButton calibrateButton;

    static final int DAPI_CONTRAST_MIN = 10;
    static final int DAPI_CONTRAST_MAX = 100;
    static final int DAPI_CONTRAST_INIT = 80;
    public JSlider DAPI_CONTRAST = new JSlider(JSlider.HORIZONTAL,
                                              DAPI_CONTRAST_MIN,
DAPI_CONTRAST_MAX, DAPI_CONTRAST_INIT);

    static final int DAPI_SENSITIVITY_MIN = 0;
    static final int DAPI_SENSITIVITY_MAX = 100;
    static final int DAPI_SENSITIVITY_INIT = 5;
    public JSlider DAPI_SENSITIVITY = new JSlider(JSlider.HORIZONTAL,
                                              DAPI_SENSITIVITY_MIN,
DAPI_SENSITIVITY_MAX, DAPI_SENSITIVITY_INIT);

    private JList destList;


    static final int RED_CONTRAST_MIN = 10;
    static final int RED_CONTRAST_MAX = 100;
    static final int RED_CONTRAST_INIT = 50;
```

```
    public JSlider RED_CONTRAST = new JSlider(JSlider.HORIZONTAL,
                                              RED_CONTRAST_MIN,
RED_CONTRAST_MAX, RED_CONTRAST_INIT);

    static final int RED_SENSITIVITY_MIN = 0;
    static final int RED_SENSITIVITY_MAX = 100;
    static final int RED_SENSITIVITY_INIT = 15;
    public JSlider RED_SENSITIVITY = new JSlider(JSlider.HORIZONTAL,
                                              RED_SENSITIVITY_MIN,
RED_SENSITIVITY_MAX, RED_SENSITIVITY_INIT);

    static final int GREEN_CONTRAST_MIN = 10;
    static final int GREEN_CONTRAST_MAX = 100;
    static final int GREEN_CONTRAST_INIT = 50;
    public JSlider GREEN_CONTRAST = new JSlider(JSlider.HORIZONTAL,
                                              GREEN_CONTRAST_MIN,
GREEN_CONTRAST_MAX, GREEN_CONTRAST_INIT);

    static final int GREEN_SENSITIVITY_MIN = 0;
    static final int GREEN_SENSITIVITY_MAX = 100;
    static final int GREEN_SENSITIVITY_INIT = 15;
    public JSlider GREEN_SENSITIVITY = new JSlider(JSlider.HORIZONTAL,
                                              GREEN_SENSITIVITY_MIN,
GREEN_SENSITIVITY_MAX, GREEN_SENSITIVITY_INIT);


    public SpinnerModel DAPI_BLUR_SPINNER_MODEL = new SpinnerNumberModel
(0,0,20,1);
    private JSpinner DAPI_BLUR_SPINNER = new JSpinner
(DAPI_BLUR_SPINNER_MODEL);

    public SpinnerModel RED_BLUR_SPINNER_MODEL = new SpinnerNumberModel
(0,0,20,1);
    private JSpinner RED_BLUR_SPINNER = new JSpinner
(RED_BLUR_SPINNER_MODEL);

    public SpinnerModel GREEN_BLUR_SPINNER_MODEL = new SpinnerNumberModel
(0,0,20,1);
    private JSpinner GREEN_BLUR_SPINNER = new JSpinner
(GREEN_BLUR_SPINNER_MODEL);


    public SpinnerModel first_peak_center_model = new SpinnerNumberModel
(0,0,300,.1);
    private JSpinner first_peak_center = new JSpinner
(first_peak_center_model);
```

```
    public SpinnerModel first_peak_lower_model = new SpinnerNumberModel
(0,0,300,.1);
    private JSpinner first_peak_lower = new JSpinner
(first_peak_lower_model);

    public SpinnerModel first_peak_upper_model = new SpinnerNumberModel
(0,0,300,.1);
    private JSpinner first_peak_upper = new JSpinner
(first_peak_upper_model);


    public int[] loaded_variables;

    static final String gapList[] = {"0", "10", "15", "20"};
    final static int maxGap = 20;

    private JRadioButton CONTRAST_MANUAL_DAPI_BUTTON = new JRadioButton
("DAPI Channel");
    private JRadioButton CONTRAST_AUTOMATIC_DAPI_BUTTON = new JRadioButton
("DAPI Channel");
    private JRadioButton CONTRAST_MANUAL_RED_BUTTON = new JRadioButton("RED
Channel");
    private JRadioButton CONTRAST_AUTOMATIC_RED_BUTTON = new JRadioButton
("RED Channel");
    private JRadioButton CONTRAST_MANUAL_GREEN_BUTTON = new JRadioButton
("GREEN Channel");
    private JRadioButton CONTRAST_AUTOMATIC_GREEN_BUTTON = new JRadioButton
("GREEN Channel");
    private JRadioButton SENSITIVITY_MANUAL_DAPI_BUTTON = new JRadioButton
("DAPI Channel");
    private JRadioButton SENSITIVITY_AUTOMATIC_DAPI_BUTTON = new
JRadioButton("DAPI Channel");
    private JRadioButton SENSITIVITY_MANUAL_RED_BUTTON = new JRadioButton
("RED Channel");
    private JRadioButton SENSITIVITY_AUTOMATIC_RED_BUTTON = new
JRadioButton("RED Channel");
    private JRadioButton SENSITIVITY_MANUAL_GREEN_BUTTON = new JRadioButton
("GREEN Channel");
    private JRadioButton SENSITIVITY_AUTOMATIC_GREEN_BUTTON = new
JRadioButton("GREEN Channel");

    public int dapi_auto_or_manual_contrast_counter;
    public int red_auto_or_manual_contrast_counter;
    public int green_auto_or_manual_contrast_counter;
    public int dapi_auto_or_manual_sensitivity_counter;
```

```
    public int red_auto_or_manual_sensitivity_counter;
    public int green_auto_or_manual_sensitivity_counter;

    private JLabel DAPI_CONTRAST_LABEL = new JLabel("Select DAPI Channel
Contrast Levels",JLabel.CENTER);
    private JLabel DAPI_SENSITIVITY_LABEL = new JLabel("Select DAPI Channel
Sensitivity Levels",JLabel.CENTER);

    private JLabel RED_CONTRAST_LABEL = new JLabel("Select RED Channel
Image Contrast Levels",JLabel.CENTER);
    private JLabel RED_SENSITIVITY_LABEL = new JLabel("Select RED Channel
Sensitivity Levels",JLabel.CENTER);

    private JLabel GREEN_CONTRAST_LABEL = new JLabel("Select GREEN Channel
Image Contrast Levels",JLabel.CENTER);
    private JLabel GREEN_SENSITIVITY_LABEL = new JLabel("Select GREEN
Channel Sensitivity Levels",JLabel.CENTER);

    private JButton loadsettingsbutton = new JButton("Load Settings
File..");
    private JButton savesettingsbutton = new JButton("Save Current
Settings");
    private JButton run_calibration    = new JButton("Calibrate..");



    public Input_Settings_Panel() {
       // this.setLayout(new GridLayout(5, 2));




        DAPI_CONTRAST.addChangeListener(new dapi_contrast_slider_change());
        RED_CONTRAST.addChangeListener(new red_contrast_slider_change());
        GREEN_CONTRAST.addChangeListener(new green_contrast_slider_change
());
        DAPI_SENSITIVITY.addChangeListener(new
dapi_sensitiity_slider_change());
        RED_SENSITIVITY.addChangeListener(new red_sensitiity_slider_change
());
        GREEN_SENSITIVITY.addChangeListener(new
green_sensitiity_slider_change());



        //Create the label.
```

```
Font font = new Font("ARIAL", Font.BOLD, 12);
DAPI_CONTRAST_LABEL.setFont(font);
DAPI_CONTRAST_LABEL.setForeground(Color.BLUE);
DAPI_SENSITIVITY_LABEL.setFont(font);
DAPI_SENSITIVITY_LABEL.setForeground(Color.BLUE);


RED_CONTRAST_LABEL.setFont(font);
RED_CONTRAST_LABEL.setForeground(Color.RED);
RED_SENSITIVITY_LABEL.setFont(font);
RED_SENSITIVITY_LABEL.setForeground(Color.RED);



GREEN_CONTRAST_LABEL.setFont(font);
GREEN_CONTRAST_LABEL.setForeground( new Color(34,139,34));
GREEN_SENSITIVITY_LABEL.setFont(font);
GREEN_SENSITIVITY_LABEL.setForeground( new Color(34,139,34));




//DAPI_CONTRAST.addChangeListener(this);


DAPI_CONTRAST.setMajorTickSpacing(10);
DAPI_CONTRAST.setMinorTickSpacing(1);
DAPI_CONTRAST.setPaintTicks(true);
DAPI_CONTRAST.setPaintLabels(true);

DAPI_SENSITIVITY.setMajorTickSpacing(10);
DAPI_SENSITIVITY.setMinorTickSpacing(1);
DAPI_SENSITIVITY.setPaintTicks(true);
DAPI_SENSITIVITY.setPaintLabels(true);

RED_CONTRAST.setMajorTickSpacing(10);
RED_CONTRAST.setMinorTickSpacing(1);
RED_CONTRAST.setPaintTicks(true);
RED_CONTRAST.setPaintLabels(true);

RED_SENSITIVITY.setMajorTickSpacing(10);
RED_SENSITIVITY.setMinorTickSpacing(1);
RED_SENSITIVITY.setPaintTicks(true);
```

```
        RED_SENSITIVITY.setPaintLabels(true);

        GREEN_CONTRAST.setMajorTickSpacing(10);
        GREEN_CONTRAST.setMinorTickSpacing(1);
        GREEN_CONTRAST.setPaintTicks(true);
        GREEN_CONTRAST.setPaintLabels(true);

        GREEN_SENSITIVITY.setMajorTickSpacing(10);
        GREEN_SENSITIVITY.setMinorTickSpacing(1);
        GREEN_SENSITIVITY.setPaintTicks(true);
        GREEN_SENSITIVITY.setPaintLabels(true);



        CONTRAST_MANUAL_DAPI_BUTTON.setForeground(Color.blue);
        CONTRAST_AUTOMATIC_DAPI_BUTTON.setForeground(Color.BLUE);

        CONTRAST_MANUAL_DAPI_BUTTON.setActionCommand("1");
        CONTRAST_MANUAL_DAPI_BUTTON.addActionListener(new
Settingsradiobutton_actionlistener());
        CONTRAST_AUTOMATIC_DAPI_BUTTON.setActionCommand("2");
        CONTRAST_AUTOMATIC_DAPI_BUTTON.addActionListener(new
Settingsradiobutton_actionlistener());


        CONTRAST_MANUAL_RED_BUTTON.setForeground(Color.RED);
        CONTRAST_AUTOMATIC_RED_BUTTON.setForeground(Color.RED);

        CONTRAST_MANUAL_RED_BUTTON.setActionCommand("3");
        CONTRAST_MANUAL_RED_BUTTON.addActionListener(new
Settingsradiobutton_actionlistener());
        CONTRAST_AUTOMATIC_RED_BUTTON.setActionCommand("4");
        CONTRAST_AUTOMATIC_RED_BUTTON.addActionListener(new
Settingsradiobutton_actionlistener());




        CONTRAST_MANUAL_GREEN_BUTTON.setForeground(new Color(34,139,34));
        CONTRAST_AUTOMATIC_GREEN_BUTTON.setForeground(new Color
(34,139,34));

        CONTRAST_MANUAL_GREEN_BUTTON.setActionCommand("5");
        CONTRAST_MANUAL_GREEN_BUTTON.addActionListener(new
Settingsradiobutton_actionlistener());
        CONTRAST_AUTOMATIC_GREEN_BUTTON.setActionCommand("6");
```

```
        CONTRAST_AUTOMATIC_GREEN_BUTTON.addActionListener(new
Settingsradiobutton_actionlistener());



        SENSITIVITY_MANUAL_DAPI_BUTTON.setForeground(Color.BLUE);
        SENSITIVITY_AUTOMATIC_DAPI_BUTTON.setForeground(Color.BLUE);

        SENSITIVITY_MANUAL_DAPI_BUTTON.setActionCommand("7");
        SENSITIVITY_MANUAL_DAPI_BUTTON.addActionListener(new
Settingsradiobutton_actionlistener());
        SENSITIVITY_AUTOMATIC_DAPI_BUTTON.setActionCommand("8");
        SENSITIVITY_AUTOMATIC_DAPI_BUTTON.addActionListener(new
Settingsradiobutton_actionlistener());


        SENSITIVITY_AUTOMATIC_RED_BUTTON.setForeground(Color.RED);
        SENSITIVITY_MANUAL_RED_BUTTON.setForeground(Color.RED);

        SENSITIVITY_MANUAL_RED_BUTTON.setActionCommand("9");
        SENSITIVITY_MANUAL_RED_BUTTON.addActionListener(new
Settingsradiobutton_actionlistener());
        SENSITIVITY_AUTOMATIC_RED_BUTTON.setActionCommand("10");
        SENSITIVITY_AUTOMATIC_RED_BUTTON.addActionListener(new
Settingsradiobutton_actionlistener());

        SENSITIVITY_MANUAL_GREEN_BUTTON.setForeground(new Color
(34,139,34));
        SENSITIVITY_AUTOMATIC_GREEN_BUTTON.setForeground(new Color
(34,139,34));


        SENSITIVITY_MANUAL_GREEN_BUTTON.setActionCommand("11");
        SENSITIVITY_MANUAL_GREEN_BUTTON.addActionListener(new
Settingsradiobutton_actionlistener());
        SENSITIVITY_AUTOMATIC_GREEN_BUTTON.setActionCommand("12");
        SENSITIVITY_AUTOMATIC_GREEN_BUTTON.addActionListener(new
Settingsradiobutton_actionlistener());




        ButtonGroup CONTRAST_BUTTONGROUP_DAPI = new ButtonGroup();
        ButtonGroup CONTRAST_BUTTONGROUP_RED = new ButtonGroup();
        ButtonGroup CONTRAST_BUTTONGROUP_GREEN = new ButtonGroup();
```

```
        ButtonGroup SENSITIVITY_BUTTONGROUP_DAPI = new ButtonGroup();
        ButtonGroup SENSITIVITY_BUTTONGROUP_RED = new ButtonGroup();
        ButtonGroup SENSITIVITY_BUTTONGROUP_GREEN = new ButtonGroup();





        //CONTRAST_AUTOMATIC_BUTTON.setSelected(true);
        //SENSITIVITY_AUTOMATIC_BUTTON.setSelected(true);

        CONTRAST_BUTTONGROUP_DAPI.add(CONTRAST_MANUAL_DAPI_BUTTON);
        CONTRAST_BUTTONGROUP_DAPI.add(CONTRAST_AUTOMATIC_DAPI_BUTTON);
        CONTRAST_BUTTONGROUP_RED.add(CONTRAST_MANUAL_RED_BUTTON);
        CONTRAST_BUTTONGROUP_RED.add(CONTRAST_AUTOMATIC_RED_BUTTON);
        CONTRAST_BUTTONGROUP_GREEN.add(CONTRAST_MANUAL_GREEN_BUTTON);
        CONTRAST_BUTTONGROUP_GREEN.add(CONTRAST_AUTOMATIC_GREEN_BUTTON);

        SENSITIVITY_BUTTONGROUP_DAPI.add(SENSITIVITY_MANUAL_DAPI_BUTTON);
        SENSITIVITY_BUTTONGROUP_DAPI.add
(SENSITIVITY_AUTOMATIC_DAPI_BUTTON);
        SENSITIVITY_BUTTONGROUP_RED.add(SENSITIVITY_MANUAL_RED_BUTTON);
        SENSITIVITY_BUTTONGROUP_RED.add(SENSITIVITY_AUTOMATIC_RED_BUTTON);
        SENSITIVITY_BUTTONGROUP_GREEN.add(SENSITIVITY_MANUAL_GREEN_BUTTON);
        SENSITIVITY_BUTTONGROUP_GREEN.add
(SENSITIVITY_AUTOMATIC_GREEN_BUTTON);

        JPanel CONTRAST_BUTTONGROUP_PANEL = new JPanel(new GridLayout(3,
1));
        Border border1 = BorderFactory.createTitledBorder(new String
("Automatic Image Contrast Settings:"));
        CONTRAST_BUTTONGROUP_PANEL.setBorder(border1);

        JPanel CONTRAST_BUTTONGROUP_PANEL2 = new JPanel(new GridLayout(3,
1));
        Border border2 = BorderFactory.createTitledBorder(new String
("Manual Image Contrast Settings:"));
        CONTRAST_BUTTONGROUP_PANEL2.setBorder(border2);

        JPanel SENSITIVITY_BUTTONGROUP_PANEL = new JPanel(new GridLayout(3,
1));
        Border border3 = BorderFactory.createTitledBorder(new String
("Automatic Image Sensitivity Settings:"));
        SENSITIVITY_BUTTONGROUP_PANEL.setBorder(border3);

        JPanel SENSITIVITY_BUTTONGROUP_PANEL2 = new JPanel(new GridLayout
```

```
(3, 1));
        Border border4 = BorderFactory.createTitledBorder(new String
("Manual Image Sensititity Settings:"));
        SENSITIVITY_BUTTONGROUP_PANEL2.setBorder(border4);



        CONTRAST_BUTTONGROUP_PANEL.add(CONTRAST_AUTOMATIC_DAPI_BUTTON);
        CONTRAST_BUTTONGROUP_PANEL2.add(CONTRAST_MANUAL_DAPI_BUTTON);
        CONTRAST_BUTTONGROUP_PANEL.add(CONTRAST_AUTOMATIC_RED_BUTTON);
        CONTRAST_BUTTONGROUP_PANEL2.add(CONTRAST_MANUAL_RED_BUTTON);
        CONTRAST_BUTTONGROUP_PANEL.add(CONTRAST_AUTOMATIC_GREEN_BUTTON);
        CONTRAST_BUTTONGROUP_PANEL2.add(CONTRAST_MANUAL_GREEN_BUTTON);

        SENSITIVITY_BUTTONGROUP_PANEL.add
(SENSITIVITY_AUTOMATIC_DAPI_BUTTON);
        SENSITIVITY_BUTTONGROUP_PANEL2.add(SENSITIVITY_MANUAL_DAPI_BUTTON);
        SENSITIVITY_BUTTONGROUP_PANEL.add
(SENSITIVITY_AUTOMATIC_RED_BUTTON);
        SENSITIVITY_BUTTONGROUP_PANEL2.add(SENSITIVITY_MANUAL_RED_BUTTON);
        SENSITIVITY_BUTTONGROUP_PANEL.add
(SENSITIVITY_AUTOMATIC_GREEN_BUTTON);
        SENSITIVITY_BUTTONGROUP_PANEL2.add
(SENSITIVITY_MANUAL_GREEN_BUTTON);

        CONTRAST_AUTOMATIC_DAPI_BUTTON.doClick();
        setdapi_contrast_automatic();

        CONTRAST_AUTOMATIC_RED_BUTTON.doClick();
        setred_contrast_automatic();

        CONTRAST_AUTOMATIC_GREEN_BUTTON.doClick();
        setgreen_contrast_automatic();

        SENSITIVITY_AUTOMATIC_DAPI_BUTTON.doClick();
        setdapi_sensitivity_automatic();

        SENSITIVITY_AUTOMATIC_RED_BUTTON.doClick();
        setred_sensitivity_automatic();

        SENSITIVITY_AUTOMATIC_GREEN_BUTTON.doClick();
        setgreen_sensitivity_automatic();


        GREEN_SENSITIVITY_LABEL.setFont(font);
        GREEN_SENSITIVITY_LABEL.setForeground( new Color(34,139,34));
```

```
        JPanel spinnerpanel = new JPanel(new GridLayout(6, 2));


        spinnerpanel.add(new JLabel("Set DAPI Channel
Blur:"));//,JLabel.CENTER);
        spinnerpanel.add(DAPI_BLUR_SPINNER);

        spinnerpanel.add(new JLabel("Set RED Channel
Blur:"));//,JLabel.CENTER);
        spinnerpanel.add(RED_BLUR_SPINNER);

        spinnerpanel.add(new JLabel("Set GREEN Channel
Blur:"));//,JLabel.CENTER);
        spinnerpanel.add(GREEN_BLUR_SPINNER);

        spinnerpanel.add(new JLabel("Set First Peak
Center:"));//,JLabel.CENTER);
        spinnerpanel.add(first_peak_center);

        spinnerpanel.add(new JLabel("Set First Peak Lower
Bound:"));//,JLabel.CENTER);
        spinnerpanel.add(first_peak_lower);

        spinnerpanel.add(new JLabel("Set First Peak Upper
Bound:"));//,JLabel.CENTER);
        spinnerpanel.add(first_peak_upper);


        JPanel automatic_or_manual_panel = new JPanel(new GridLayout(2,
2));



        automatic_or_manual_panel.add(CONTRAST_BUTTONGROUP_PANEL);
        automatic_or_manual_panel.add(CONTRAST_BUTTONGROUP_PANEL2);


        automatic_or_manual_panel.add(SENSITIVITY_BUTTONGROUP_PANEL);
        automatic_or_manual_panel.add(SENSITIVITY_BUTTONGROUP_PANEL2);

        JPanel Input_settings_top_panel = new JPanel(new GridLayout(1, 2,
50,0));
        Input_settings_top_panel.add(spinnerpanel, BorderLayout.WEST);
        Input_settings_top_panel.add(automatic_or_manual_panel,
BorderLayout.EAST);
```

```
        savesettingsbutton.addActionListener(new savefilebutton_action());
        loadsettingsbutton.addActionListener(new loadfilebutton_action());
        run_calibration.addActionListener(new run_calibration_action());


        JPanel slidersL = new JPanel(new GridLayout(6,1));
        JPanel slidersC = new JPanel(new GridLayout(6,1));
        JPanel slidersR = new JPanel(new GridLayout(6,1));
        JPanel combination_panel = new JPanel(new GridLayout(1,3,50,0));




        slidersL.add(DAPI_CONTRAST_LABEL);
        slidersL.add(DAPI_CONTRAST);
        slidersL.add(RED_CONTRAST_LABEL);
        slidersL.add(RED_CONTRAST);
        slidersL.add(GREEN_CONTRAST_LABEL);
        slidersL.add(GREEN_CONTRAST);

        slidersR.add(DAPI_SENSITIVITY_LABEL);
        slidersR.add(DAPI_SENSITIVITY);
        slidersR.add(RED_SENSITIVITY_LABEL);
        slidersR.add(RED_SENSITIVITY);
        slidersR.add(GREEN_SENSITIVITY_LABEL);
        slidersR.add(GREEN_SENSITIVITY);

        slidersC.add(new JLabel("         "));
        slidersC.add(loadsettingsbutton);
        slidersC.add(new JLabel("         "));
        slidersC.add(savesettingsbutton);
        slidersC.add(new JLabel("         "));
        slidersC.add(run_calibration);
//        slidersC.add(new JLabel("         "));
//        slidersC.add(new JLabel("         "));
//        slidersC.add(new JLabel("         "));

        combination_panel.add(slidersL);
        combination_panel.add(slidersC);
        combination_panel.add(slidersR);

        //Load file buttons and panel
```

```
        JPanel loadfilepanel = new JPanel();
        JPanel wholepanel = new JPanel(new GridLayout(2,1));//BorderLayout
());


//        loadfilepanel.add(loadsettingsbutton);
//        loadfilepanel.add(savesettingsbutton);
//        loadfilepanel.add(run_calibration);

        wholepanel.add(Input_settings_top_panel);//, BorderLayout.NORTH);
        wholepanel.add(combination_panel);//,BorderLayout.CENTER);
        //wholepanel.add(loadfilepanel,BorderLayout.SOUTH);



        this.add(wholepanel);

    }


       public int getCONTRAST_DAPI(){
      return this.DAPI_CONTRAST.getValue();
  }
        public int getCONTRAST_RED()
  {
     return this.RED_CONTRAST.getValue();
  }
        public int getCONTRAST_GREEN()
  {
     return this.GREEN_CONTRAST.getValue();
  }
        public int getSENSITIVITY_DAPI()
  {
     return DAPI_SENSITIVITY.getValue();
  }
        public int getSENSITIVITY_RED()
  {
     return RED_SENSITIVITY.getValue();
  }
        public int getSENSITIVITY_GREEN()
  {
     return GREEN_SENSITIVITY.getValue();
  }
        public int getBLUR_DAPI()
  {
     return (Integer) DAPI_BLUR_SPINNER.getValue();
```

```
}
      public int getBLUR_RED()
{
   return (Integer) RED_BLUR_SPINNER.getValue();
}
      public int getBLUR_GREEN()
{
   return (Integer) GREEN_BLUR_SPINNER.getValue();
}
       public double getfpc()
{
   return (Double) first_peak_center.getValue();
}
       public double getfpu()
{
   return (Double) first_peak_upper.getValue();
}
       public double getfpl()
{
   return (Double) first_peak_lower.getValue();
}
```

```
      public int[] getAUTOMAN(){
      int[] auto_man = new int[6];
       auto_man[0] = dapi_auto_or_manual_contrast_counter;
       auto_man[1] = red_auto_or_manual_contrast_counter;
       auto_man[2] = green_auto_or_manual_contrast_counter;
       auto_man[3] = dapi_auto_or_manual_sensitivity_counter;
       auto_man[4] = red_auto_or_manual_sensitivity_counter;
       auto_man[5] = green_auto_or_manual_sensitivity_counter;

          return auto_man;
      }
```

```
       protected class Settingsradiobutton_actionlistener implements
ActionListener {
        public void actionPerformed(ActionEvent e) {


         String actionC = e.getActionCommand();
         int casex = Integer.parseInt(actionC);
                        //System.out.println(casex);
                        //System.out.println(actionC);


         switch (casex){
             case 1:
               //  System.out.println("Manual DapiC");
                 dapi_auto_or_manual_contrast_counter = 0;
                 setdapi_contrast_manual();
                 break;
             case 2:
               //  System.out.println("Auto DapiC");
                 dapi_auto_or_manual_contrast_counter = 1;
                 setdapi_contrast_automatic();
                 break;

             case 3: //System.out.println("Manual redC");
             red_auto_or_manual_contrast_counter = 0;
             setred_contrast_manual();
             break;
             case 4: //System.out.println("Auto redC");
             red_auto_or_manual_contrast_counter = 1;
             setred_contrast_automatic();
             break;
             case 5:// System.out.println("Manual greenC");
             green_auto_or_manual_contrast_counter = 0;
             setgreen_contrast_manual();
             break;
```

```
            case 6: //System.out.println("Auto greenC");
            green_auto_or_manual_contrast_counter = 1;
            setgreen_contrast_automatic();
            break;
            case 7: //System.out.println("Manual DapiS");
            dapi_auto_or_manual_sensitivity_counter = 0;
            setdapi_sensitivity_manual();
            break;
            case 8: //System.out.println("Auto DapiS");
            dapi_auto_or_manual_sensitivity_counter = 1;
            setdapi_sensitivity_automatic();
            break;
            case 9: //System.out.println("Manual redS");
            red_auto_or_manual_sensitivity_counter = 0;
            setred_sensitivity_manual();
            break;
            case 10:// System.out.println("Auto redS");
            red_auto_or_manual_sensitivity_counter = 1;
            setred_sensitivity_automatic();

            break;
            case 11: //System.out.println("Manual greenS");
            green_auto_or_manual_sensitivity_counter = 0;
            setgreen_sensitivity_manual();

            break;
            case 12: //System.out.println("Auto greenS");
            green_auto_or_manual_sensitivity_counter = 1;
            setgreen_sensitivity_automatic();

            break;

          }

 }
    }



public void setdapi_contrast_automatic(){
    DAPI_CONTRAST.setEnabled(false);
}

public void setdapi_contrast_manual(){
    DAPI_CONTRAST.setEnabled(true);
}
```

```
public void setred_contrast_automatic(){
    RED_CONTRAST.setEnabled(false);

}

public void setred_contrast_manual(){
    RED_CONTRAST.setEnabled(true);

}

public void setgreen_contrast_automatic(){
    GREEN_CONTRAST.setEnabled(false);
}

public void setgreen_contrast_manual(){
    GREEN_CONTRAST.setEnabled(true);

}

public void setdapi_sensitivity_automatic(){
    DAPI_SENSITIVITY.setEnabled(false);
}

public void setdapi_sensitivity_manual(){
    DAPI_SENSITIVITY.setEnabled(true);
}


public void setred_sensitivity_automatic(){
    RED_SENSITIVITY.setEnabled(false);
}

public void setred_sensitivity_manual(){
    RED_SENSITIVITY.setEnabled(true);
}

public void setgreen_sensitivity_automatic(){
    GREEN_SENSITIVITY.setEnabled(false);
}

public void setgreen_sensitivity_manual(){
    GREEN_SENSITIVITY.setEnabled(true);
}
```

```
         protected class savefilebutton_action implements ActionListener {

    public void actionPerformed(ActionEvent e) {
     //   System.out.println("Action Listner Triggered, SAVE file is being 
pushed");


          int DAPI_CONTRAST2  = getCONTRAST_DAPI();
          int RED_CONTRAST2   = getCONTRAST_RED();
          int GREEN_CONTRAST2 = getCONTRAST_GREEN();

          int DAPI_SENSITIVITY2 = getSENSITIVITY_DAPI();
          int RED_SENSITIVITY2   =  getSENSITIVITY_RED();
          int GREEN_SENSITIVITY2  = getSENSITIVITY_GREEN();
          int DAPI_BLUR_SPINNER2  = getBLUR_DAPI();
          int RED_BLUR_SPINNER2   = getBLUR_RED();
          int GREEN_BLUR_SPINNER2 = getBLUR_GREEN();
          double fpc = getfpc();
          double fpu = getfpu();
          double fpl = getfpl();


      String file_name = null;
      JFileChooser fileChooser = new JFileChooser(System.getProperty
("user.dir"));
      fileChooser.setDialogTitle("Save Settings File");
      int returnVal = fileChooser.showSaveDialog(null);
      if(returnVal == fileChooser.APPROVE_OPTION){
               file_name = fileChooser.getSelectedFile().toString()
+".txt";
      }


        Settings_File_Loader filesave = new Settings_File_Loader();
        if(file_name!=null){
        filesave.savefile
(file_name,DAPI_CONTRAST2,RED_CONTRAST2,GREEN_CONTRAST2,DAPI_SENSITIVITY2,

RED_SENSITIVITY2,GREEN_SENSITIVITY2,DAPI_BLUR_SPINNER2,RED_BLUR_SPINNER2,GR
EEN_BLUR_SPINNER2,fpc,fpl,fpu,

dapi_auto_or_manual_contrast_counter,red_auto_or_manual_contrast_counter,gr
een_auto_or_manual_contrast_counter,
```

```
dapi_auto_or_manual_sensitivity_counter,red_auto_or_manual_sensitivity_coun
ter,green_auto_or_manual_sensitivity_counter);

        }



    }

  }

         protected class loadfilebutton_action implements ActionListener {

    public void actionPerformed(ActionEvent e) {

     //   System.out.println("Action Listner Triggered, LOAD file is being
pushed");
      String file_name = null;
      Settings_File_Loader filesave = new Settings_File_Loader();
      JFileChooser directory_chooser = new JFileChooser(System.getProperty
("user.dir"));

      directory_chooser.setDialogTitle("Load settings file (TXT");
      directory_chooser.setAcceptAllFileFilterUsed(false);

     if (directory_chooser.showOpenDialog(null) ==
JFileChooser.APPROVE_OPTION) {
      file_name = directory_chooser.getSelectedFile().toString();
     }
     if(file_name != null){

        double[] ints = (double[])filesave.loadfile(file_name);

        DAPI_CONTRAST.setValue((int) ints[0]);
        RED_CONTRAST.setValue((int)ints[1]);
        GREEN_CONTRAST.setValue((int)ints[2]);
        DAPI_SENSITIVITY.setValue((int)ints[3]);
        RED_SENSITIVITY.setValue((int)ints[4]);
        GREEN_SENSITIVITY.setValue((int)ints[5]);
        DAPI_BLUR_SPINNER.setValue((int)ints[6]);
        RED_BLUR_SPINNER.setValue((int)ints[7]);
        GREEN_BLUR_SPINNER.setValue((int)ints[8]);
        first_peak_center.setValue(ints[9]);
        first_peak_lower.setValue(ints[10]);
        first_peak_upper.setValue(ints[11]);
```

```
        if((int)ints[12] == 0){
            CONTRAST_MANUAL_DAPI_BUTTON.doClick();
        }else{
            CONTRAST_AUTOMATIC_DAPI_BUTTON.doClick();
        }

        if((int)ints[13] == 0){
            CONTRAST_MANUAL_RED_BUTTON.doClick();
        }else{
            CONTRAST_AUTOMATIC_RED_BUTTON.doClick();
        }

        if((int)ints[14] == 0){
            CONTRAST_MANUAL_GREEN_BUTTON.doClick();
        }else{
            CONTRAST_AUTOMATIC_GREEN_BUTTON.doClick();
        }

        if((int)ints[15] == 0){
            SENSITIVITY_MANUAL_DAPI_BUTTON.doClick();
        }else{
            SENSITIVITY_AUTOMATIC_DAPI_BUTTON.doClick();
        }

        if((int)ints[16] == 0){
            SENSITIVITY_MANUAL_RED_BUTTON.doClick();
        }else{
            SENSITIVITY_AUTOMATIC_RED_BUTTON.doClick();
        }

        if((int)ints[17] == 0){
            SENSITIVITY_MANUAL_GREEN_BUTTON.doClick();
        }else{
            SENSITIVITY_AUTOMATIC_GREEN_BUTTON.doClick();
        }
    }
         }
  }




         protected class dapi_contrast_slider_change implements
ChangeListener {
```

```
    public void stateChanged(ChangeEvent e) {
        //System.out.println("Slider change event registered");
        JSlider source = (JSlider)e.getSource();


        int slidervalue = (int)source.getValue();
        DAPI_CONTRAST_LABEL.setText("Select DAPI Channel Contrast Levels:
Current Level: "+slidervalue);

        if (!source.getValueIsAdjusting()) {
            int DAPI_CONTRAST2  = getCONTRAST_DAPI();
        //    System.out.println("getContrastDapi" + DAPI_CONTRAST2);
       }
   }
        }



         protected class red_contrast_slider_change implements
ChangeListener {
    public void stateChanged(ChangeEvent e) {
        //System.out.println("Slider change event registered");
        JSlider source = (JSlider)e.getSource();
        int slidervalue = (int)source.getValue();
        RED_CONTRAST_LABEL.setText("Select RED Channel Image Contrast
Levels:  Current Level: "+slidervalue);
        if (!source.getValueIsAdjusting()) {
            int RED_CONTRAST2  = getCONTRAST_RED();
        //    System.out.println("getContrastRED" + RED_CONTRAST2);
       }
   }
        }

         protected class green_contrast_slider_change implements
ChangeListener {
    public void stateChanged(ChangeEvent e) {
        //System.out.println("Slider change event registered");
        JSlider source = (JSlider)e.getSource();
        int slidervalue = (int)source.getValue();
        GREEN_CONTRAST_LABEL.setText("Select GREEN Channel Image Contrast
Levels:  Current Level: "+slidervalue);
        if (!source.getValueIsAdjusting()) {
            int GREEN_CONTRAST2  = getCONTRAST_GREEN();
        //    System.out.println("getContrastGREEN" + GREEN_CONTRAST2);
       }
   }
```

```
        }



    protected class dapi_sensitiity_slider_change implements ChangeListener
{
    public void stateChanged(ChangeEvent e) {
        //System.out.println("Slider change event registered");

        JSlider source = (JSlider)e.getSource();
        int slidervalue = (int)source.getValue();
        DAPI_SENSITIVITY_LABEL.setText("Select DAPI Channel Sensitivity
Levels:  Current Level: "+slidervalue);
        if (!source.getValueIsAdjusting()) {
            int SENSITIVITY_DAPI2  = getSENSITIVITY_DAPI();

        //    System.out.println("getSensitivityDapi" + SENSITIVITY_DAPI2);
    }

   }
   }

    protected class red_sensitiity_slider_change implements ChangeListener
{
    public void stateChanged(ChangeEvent e) {
      //  System.out.println("Slider change event registered");

        JSlider source = (JSlider)e.getSource();
        int slidervalue = (int)source.getValue();
        RED_SENSITIVITY_LABEL.setText("Select RED Channel Sensitivity
Levels:  Current Level: "+slidervalue);

        if (!source.getValueIsAdjusting()) {
            int SENSITIVITY_RED2  = getSENSITIVITY_RED();

         //   System.out.println("getSensitivityRED" + SENSITIVITY_RED2);
    }

   }
   }

    protected class green_sensitiity_slider_change implements
ChangeListener {
    public void stateChanged(ChangeEvent e) {
        //System.out.println("Slider change event registered");
```

```
        JSlider source = (JSlider)e.getSource();
        int slidervalue = (int)source.getValue();
        GREEN_SENSITIVITY_LABEL.setText("Select GREEN Channel Sensitivity 
Levels:  Current Level: "+slidervalue);

        if (!source.getValueIsAdjusting()) {
            int SENSITIVITY_GREEN2  = getSENSITIVITY_GREEN();

        //    System.out.println("getSensitivityGreen" + 
SENSITIVITY_GREEN2);
    }

   }
   }


    public JButton getsavebutton(){
        return savesettingsbutton;
    }

     public JButton getloadbutton(){
        return loadsettingsbutton;
    }




      protected class run_calibration_action implements ActionListener {

    public void actionPerformed(ActionEvent e) {


     if(processButton != null){
     calibrateButton.doClick();
     }

      if(processButton == null){
         System.out.println("Please Load a Dataset to Calibrate...");
      }


  }




      }
```

```
      public void placedir(JButton button, JButton button2, JList list1){
        processButton = button;
        calibrateButton = button2;
        destList = list1;

    }

      public JList getDestList(){
        return destList;
    }

      public JButton setCalibrationButton(){
          return run_calibration;
      }




}
```
