# Supplementary material for: Satellite cell heterogeneity revealed by G-Tool, an open algorithm to quantify myogenesis through colony-forming assays
Source: Skelet Muscle. 2012 Jun 15;2:13. doi: 10.1186/2044-5040-2-13 (PMC3439689; doi:10.1186/2044-5040-2-13)
Supplement: Additional file 1 — G-Tool Source Code. Java and MATLAB Source Codes are included. [file 2044-5040-2-13-S1.zip › G-Tool Sourcecode and PDF files/PDF files of code/JAVA - GUI/MainProgram.pdf]

```
/*%     This file is part of GTOOL. AUTHOR: JOSEPH IPPOLITO, THE UNIVERSITY
%       OF MINNESOTA. GTOOL is free software: you can redistribute it
%   and/or modify
%       it under the terms of the GNU General Public License as published
%   by the Free Software Foundation, either version 3 of the License, or
%       (at your option) any later version.
%       GTOOL is distributed in the hope that it will be useful,
%   but WITHOUT ANY WARRANTY; without even the implied warranty of
%   MERCHANTABILITY or FITNESS FOR A PARTICULAR PURPOSE. SEE THE GNU
%   GENERAL PUBLIC LISCENCE FOR MORE DETAILS.
%       You should have received a copy of the GNU General Public License
%   along with GTOOL. If not see see <http://www.gnu.org/licenses/>. */
package gtool;

import gtoolProject.*;
import java.util.*;
import java.awt.event.*;
import com.mathworks.toolbox.javabuilder.*;
import java.util.logging.Level;
import java.util.logging.Logger;
import java.lang.*;
import java.awt.*;
import java.awt.image.*;
import java.io.*;
import javax.swing.*;
import java.util.ArrayList;


public class MainProgram extends JPanel {
    private Calendar calendar = new GregorianCalendar();
    private Menubar menubar2 = new Menubar();

    private final Image_Selection_Panel image_panel = new
Image_Selection_Panel();
    private final Input_Settings_Panel settingspanelpanel = new
Input_Settings_Panel();
    private final Output_Settings_Panel outputpanelsettingspanel = new
Output_Settings_Panel();
    private JTextArea textArea;
    private JList destination_list;

    private JTabbedPane tabbedPane = new JTabbedPane();
    private File dir = new java.io.File("C:\\Documents and Settings\\Jack\
\Desktop\\Human vs Computer counting\\Images\\Bl6");

    private JPanel datapanel = new JPanel(new BorderLayout());
```

```
    private JPanel imagepanel = new JPanel(new BorderLayout());
    private JPanel settingspanel = new JPanel();
    private JPanel outputpanel = new JPanel();
    private JPanel visualpanel = new JPanel();
    private JPanel buttonPaneImageDir = new JPanel();
    private JLabel directorylabel = new JLabel("No Directory Selected");
    private JLabel output_label1 = new JLabel("starting");
    private JButton processButton = new JButton("Process Images");
    private JButton calibrateButton = new JButton("Calibrate");

    private JLabel image_output_label = new JLabel("",JLabel.CENTER);
    private JPanel test_output_panel = new JPanel();
    private JPanel display_output_panel = new JPanel();
    private JPanel consolePanel = new JPanel(new BorderLayout());
    private BufferedImage img;
    private JProgressBar progressBar;
    private int hour = calendar.get(Calendar.HOUR);
    private int date = calendar.get(Calendar.DATE);
    private int month = calendar.get(Calendar.MONTH);
    private File trace_directory = null;

    public MainProgram() {
       super(new GridLayout(1, 1));

        //////////////////
        tabbedPane.addTab("Input Settings", null, settingspanel);
        settingspanel.setLayout(new BorderLayout());
        settingspanel.add(settingspanelpanel,BorderLayout.CENTER);


        tabbedPane.addTab("OutputSettings", null, outputpanel);
        outputpanel.setLayout(new BorderLayout());
        outputpanel.add(outputpanelsettingspanel,BorderLayout.CENTER);


        //////////////////



        tabbedPane.addTab("Add Images", null, imagepanel);
        JButton selectDirectoryButton = new JButton("Select Image
Directory");
        selectDirectoryButton.addActionListener(new selectDirectoryListener
());

        processButton.addActionListener(new processImages());
        calibrateButton.addActionListener(new calibrateImages());
```

```
        buttonPaneImageDir.setLayout(new BoxLayout(buttonPaneImageDir,
BoxLayout.LINE_AXIS));
        buttonPaneImageDir.setBorder(BorderFactory.createEmptyBorder(0, 10,
10, 10));

        buttonPaneImageDir.add(directorylabel,BorderLayout.EAST);
        buttonPaneImageDir.add(Box.createHorizontalGlue());
        buttonPaneImageDir.add(selectDirectoryButton);
        buttonPaneImageDir.add(Box.createRigidArea(new Dimension(10, 0)));
        buttonPaneImageDir.add(processButton);
        processButton.setEnabled(false);


        imagepanel.add(buttonPaneImageDir,BorderLayout.PAGE_END);
        imagepanel.add(image_panel, BorderLayout.WEST);

        imagepanel.setSize(400, 300);

        //DATA PANEL
        progressBar = new JProgressBar();
        textArea = new JTextArea(5, 50);
        JScrollPane textscrollPane = new JScrollPane(textArea);
        //textArea.setEditable(false);

        textArea.append("");

        redirectSystemStreams();
        consolePanel.add(textscrollPane,BorderLayout.CENTER);
        tabbedPane.addTab("Console",null,consolePanel);


        add(tabbedPane);
        tabbedPane.setTabLayoutPolicy(JTabbedPane.SCROLL_TAB_LAYOUT);
        tabbedPane.setSelectedComponent(imagepanel);

    }



     public JTabbedPane getTabbedPane()
  {
     return tabbedPane;
  }
```

```
   public JPanel getTab1()
  {
    return settingspanel;
  }

   public JPanel getTab2()
  {
    return imagepanel;
  }
   public JPanel getTab3()
  {
    return datapanel;
  }

   public JPanel getTab4()
  {
    return visualpanel;
  }

   public  File getDir()
  {
    return dir;
  }




    protected class selectDirectoryListener implements ActionListener {


        @Override
    public void actionPerformed(ActionEvent e) {
      int xlt;
      File comparison = dir;

      JFileChooser directory_chooser = new JFileChooser();
    //  System.out.println("getCurrentDirectory(): " +
directory_chooser.getCurrentDirectory());
      //directory_chooser.setCurrentDirectory(dir);
      directory_chooser.setDialogTitle("Please Select Your Image
Directory");
      directory_chooser.setFileSelectionMode
(JFileChooser.DIRECTORIES_ONLY);
      directory_chooser.setAcceptAllFileFilterUsed(false);
```

```
     if (directory_chooser.showOpenDialog(null) ==
JFileChooser.APPROVE_OPTION) {
     // System.out.println("getSelectedFile() : " +
directory_chooser.getSelectedFile());

      //System.out.println("dir before :" + dir);

     dir = directory_chooser.getSelectedFile(); //reset the directory with
current to make it easier
     directorylabel.setText(dir.toString());


     if(comparison.compareTo(dir) == 1 ){
     xlt = 1;
      } else{
     image_panel.clearSourceListModel();
     image_panel.clearDestinationListModel();
     comparison = dir;
     xlt = 0;
      }

    String imagespath = directory_chooser.getSelectedFile().toString();

    image_panel.placedir
(dir,imagepanel,output_label1,test_output_panel,processButton,calibrateButt
on);


    File folder = new File(imagespath);
    File[] listOfFiles = folder.listFiles();

    ArrayList imagesindirectory = new ArrayList();

    for (int i = 0; i < listOfFiles.length; i++)
      {
       if (listOfFiles[i].isFile())
       {
       String files = listOfFiles[i].getName();


       if (files.endsWith(".jpg") || files.endsWith(".JPG") ||
files.endsWith(".Jpg")||files.endsWith(".jPG")||files.endsWith(".jpG") ||
files.endsWith(".bmp") || files.endsWith(".BMP") ||files.endsWith(".png")||
files.endsWith(".PNG")||files.endsWith(".JPEG")||files.endsWith(".jpeg")||
files.endsWith(".Jpeg")||files.endsWith(".jPEG"))
```

```
        {
          imagesindirectory.add(files);
        }
        }
       }


    String[] imagesindir2 = new String[imagesindirectory.size()];
    imagesindirectory.toArray(imagesindir2);
    if(xlt == 0){
        image_panel.addSourceElements(imagesindir2);
                }
    }
  }
}

    protected class processImages implements ActionListener {
        @Override
    public void actionPerformed(ActionEvent e) {

       String button_time = String.valueOf(System.currentTimeMillis());
       Runtime r = Runtime.getRuntime();
       r.gc();


       tabbedPane.setSelectedComponent(consolePanel);
       tabbedPane.revalidate();

       progressBar.setIndeterminate(true);
       destination_list = image_panel.getDestList();
       int list_size = destination_list.getModel().getSize();
       String image_path_name;
       System.out.println("Number of images in Processing Queue: " +
list_size);
       //JTable table = dataPanelinfopanel.getTable();

       int dapi_sensitivity   = settingspanelpanel.getSENSITIVITY_DAPI();
       int red_sensitivity   =  settingspanelpanel.getSENSITIVITY_RED();
       int green_sensitivity  = settingspanelpanel.getSENSITIVITY_GREEN();

       double fpc = settingspanelpanel.getfpc();
       double fpl = settingspanelpanel.getfpl();
       double fpu = settingspanelpanel.getfpu();


       int[] auto_man = settingspanelpanel.getAUTOMAN();
```

```
       int selected_channel = 3;

       int contrast_threshold       = settingspanelpanel.getCONTRAST_DAPI
();
       int contrast_threshold_red   = settingspanelpanel.getCONTRAST_RED();
       int contrast_threshold_green = settingspanelpanel.getCONTRAST_GREEN
();

              counting_program alpha = null;
            try {
                alpha = new counting_program();
            } catch (MWException ex) {
                Logger.getLogger(MainProgram.class.getName()).log
(Level.SEVERE, null, ex);
            }

       ArrayList output_settings =  outputpanelsettingspanel.getSettings();
       int trace_save_options  = Integer.parseInt(output_settings.get
(0).toString());//DONE
//       0 SettingsList.add(save_trace_counter);      0/1
      int data_save_option = Integer.parseInt(output_settings.get
(2).toString()); //DONE
//       2 SettingsList.add(data_options_counter);    1/2/3

      int nuclear_stain_option = Integer.parseInt(output_settings.get
(3).toString()); //DONE
//       3 SettingsList.add(Nuclear_Stain_counter);   1/2/3/4
      int primary_stain_option = Integer.parseInt(output_settings.get
(4).toString()); //DONE
//       4 SettingsList.add(Primary_Stain_counter);   1/2/3/4
      int secondary_stain_option = Integer.parseInt(output_settings.get
(5).toString()); //DONE
//       5 SettingsList.add(Secondary_Stain_counter); 1/2/3/4
      int counting_options = Integer.parseInt(output_settings.get
(6).toString()); //DONE
//       6 SettingsList.add(counting_options_counter); 0/1

      Integer[] stain_selection = new Integer[4];
      stain_selection[0] = nuclear_stain_option;
      stain_selection[1] = primary_stain_option;
      stain_selection[2] = secondary_stain_option;
      stain_selection[3] = counting_options;
```

```
int blur_factorD = settingspanelpanel.getBLUR_DAPI();
int blur_factorP = settingspanelpanel.getBLUR_RED();
int blur_factorS = settingspanelpanel.getBLUR_GREEN();

int contrast_threshold_primary = 0;
int contrast_threshold_secondary = 0;
int disk1 = 0;
int disk2 = 0;
int disk3 = 0;

disk1 = dapi_sensitivity;


switch (primary_stain_option){
       case 1:
           System.out.println("Primary Stain: RED");
           contrast_threshold_primary = contrast_threshold_red;
           disk2 = red_sensitivity;
           blur_factorP = settingspanelpanel.getBLUR_RED();

           break;
       case 2:
           System.out.println("Primary Stain: Green");
           contrast_threshold_primary = contrast_threshold_green;
           disk2 = green_sensitivity;
           blur_factorP = settingspanelpanel.getBLUR_GREEN();

           break;
       case 3:
           System.out.println("Primary Stain: Blue");
           contrast_threshold_primary = contrast_threshold;
           disk2 = dapi_sensitivity;
           blur_factorP = settingspanelpanel.getBLUR_DAPI();

           break;
 }

 switch (secondary_stain_option){
       case 1:
           System.out.println("Secondary Stain: RED");
           contrast_threshold_secondary = contrast_threshold_red;
           disk3 = red_sensitivity;
           blur_factorS = settingspanelpanel.getBLUR_RED();
           break;
       case 2:
           System.out.println("Secondary Stain: Green");
```

```
                contrast_threshold_secondary = contrast_threshold_green;
                disk3 = green_sensitivity;
                blur_factorS = settingspanelpanel.getBLUR_GREEN();


                break;
            case 3:
                System.out.println("Secondary Stain: Blue");
                contrast_threshold_secondary = contrast_threshold;
                disk3 = dapi_sensitivity;
                blur_factorS = settingspanelpanel.getBLUR_DAPI();

                break;
      }


      double first_peak_center = fpc;
      double first_peak_lower = fpl/fpc;
      double first_peak_upper = fpu/fpc;


      progressBar.setValue(0);

      String[] image_list = new String[list_size];

               for(int i = 0; i<list_size ;i++) //Main loop through Images
             {
                  image_path_name = dir + "\\" + destination_list.getModel
().getElementAt(i).toString();
                   image_list[i] = image_path_name;
             }


      Object[] result = null;

      int debug = 0;



            try {
                              //counting_program
(8,stain_selection,image_directory,image_list,blur_factor,blur_factorPrimar
y,blur_factorSecondary,disk1,disk2,disk3,contrast_threshold1,contrast_thres
hold2,contrast_threshold3,first_peak_center,first_peak_lower,first_peak_upp
er,plot_or_not,data_save,debug)
               result = alpha.counting_program
```

```
(8,stain_selection,dir.toString
(),image_list,blur_factorD,blur_factorP,blur_factorS,disk1,disk2,disk3,cont
rast_threshold,contrast_threshold_primary,contrast_threshold_secondary,firs
t_peak_center,first_peak_lower,first_peak_upper,trace_save_options,data_sav
e_option,debug);
            } catch (MWException ex) {
                Logger.getLogger(MainProgram.class.getName()).log
(Level.SEVERE, null, ex);
            }



    }

    }



    protected class calibrateImages implements ActionListener {
        @Override
    public void actionPerformed(ActionEvent e) {


       tabbedPane.setSelectedComponent(consolePanel);
       tabbedPane.revalidate();

       progressBar.setIndeterminate(true);
       destination_list = image_panel.getDestList();
       int list_size = destination_list.getModel().getSize();
       String image_path_name;
       System.out.println("Number of images in Calibration Queue: " +
list_size);

       int contrast_threshold1       = settingspanelpanel.getCONTRAST_DAPI
();
       int disk1 = settingspanelpanel.getSENSITIVITY_DAPI();
       int blur_factorD = settingspanelpanel.getBLUR_DAPI();;

       calibration zed = null;

       try {
                zed = new calibration();
            }catch (MWException ex) {
                Logger.getLogger(MainProgram.class.getName()).log
(Level.SEVERE, null, ex);
            }
```

```
        progressBar.setValue(0);
        String[] image_list = new String[list_size];
        Object[] result0 = null;

                 for(int i = 0; i<list_size ;i++) //Main loop through Images
               {
                    image_path_name = dir + "\\" + destination_list.getModel
().getElementAt(i).toString();
                     image_list[i] = image_path_name;
                }

             try {
                 result0 = zed.calibration
(1,image_list,blur_factorD,disk1,contrast_threshold1);

             } catch (MWException ex) {
                 Logger.getLogger(MainProgram.class.getName()).log
(Level.SEVERE, null, ex);
             }


        //joblabel.setText("Job List: Calibration Finished. " + (list_size)
+ " Images Parsed");
        progressBar.setIndeterminate(false);


    }

        }


    private void updateTextArea(final String text) {
  SwingUtilities.invokeLater(new Runnable() {
    public void run() {
      textArea.append(text);
    }
  });
}

private void redirectSystemStreams() {
  OutputStream out = new OutputStream() {
    @Override
    public void write(int b) throws IOException {
      updateTextArea(String.valueOf((char) b));
```

```
    }

    @Override
    public void write(byte[] b, int off, int len) throws IOException {
      updateTextArea(new String(b, off, len));
    }

    @Override
    public void write(byte[] b) throws IOException {
      write(b, 0, b.length);
    }
  };

  System.setOut(new PrintStream(out, true));
  System.setErr(new PrintStream(out, true));
}

  public static BufferedImage toBufferedImage(Image image) {
    if (image instanceof BufferedImage) {
        return (BufferedImage)image;
    }


    // This code ensures that all the pixels in the image are loaded
    image = new ImageIcon(image).getImage();

    // Determine if the image has transparent pixels; for this method's
    // implementation, see Determining If an Image Has Transparent Pixels
    boolean hasAlpha = false;

    // Create a buffered image with a format that's compatible with the
screen
    BufferedImage bimage = null;
    GraphicsEnvironment ge =
GraphicsEnvironment.getLocalGraphicsEnvironment();
    try {
        // Determine the type of transparency of the new buffered image
        int transparency = Transparency.OPAQUE;
        if (hasAlpha) {
            transparency = Transparency.BITMASK;
        }

        // Create the buffered image
        GraphicsDevice gs = ge.getDefaultScreenDevice();
        GraphicsConfiguration gc = gs.getDefaultConfiguration();
        bimage = gc.createCompatibleImage(
```

```
            image.getWidth(null), image.getHeight(null), transparency);
    } catch (HeadlessException e) {
        // The system does not have a screen
    }

    if (bimage == null) {
        // Create a buffered image using the default color model
        int type = BufferedImage.TYPE_INT_RGB;
        if (hasAlpha) {
            type = BufferedImage.TYPE_INT_ARGB;
        }
        bimage = new BufferedImage(image.getWidth(null), image.getHeight
(null), type);
    }

    // Copy image to buffered image
    Graphics g = bimage.createGraphics();

    // Paint the image onto the buffered image
    g.drawImage(image, 0, 0, null);
    g.dispose();
    return bimage;
}
}
```
