# Supplementary material for: Satellite cell heterogeneity revealed by G-Tool, an open algorithm to quantify myogenesis through colony-forming assays
Source: Skelet Muscle. 2012 Jun 15;2:13. doi: 10.1186/2044-5040-2-13 (PMC3439689; doi:10.1186/2044-5040-2-13)
Supplement: Additional file 1 — G-Tool Source Code. Java and MATLAB Source Codes are included. [file 2044-5040-2-13-S1.zip › G-Tool Sourcecode and PDF files/PDF files of code/JAVA - GUI/Menubar.pdf]

```
/*%     This file is part of GTOOL. AUTHOR: JOSEPH IPPOLITO, THE UNIVERSITY
%       OF MINNESOTA. GTOOL is free software: you can redistribute it
%   and/or modify
%       it under the terms of the GNU General Public License as published
%   by the Free Software Foundation, either version 3 of the License, or
%       (at your option) any later version.
%       GTOOL is distributed in the hope that it will be useful,
%   but WITHOUT ANY WARRANTY; without even the implied warranty of
%   MERCHANTABILITY or FITNESS FOR A PARTICULAR PURPOSE. SEE THE GNU
%   GENERAL PUBLIC LISCENCE FOR MORE DETAILS.
%       You should have received a copy of the GNU General Public License
%   along with GTOOL. If not see see <http://www.gnu.org/licenses/>. */
package gtool;
import java.awt.event.*;
import javax.swing.*;

public class Menubar{
    private JMenuItem settingsmenuItem = new JMenuItem("Import Settings to
File");
    private JMenuItem analyzesmenuItem = new JMenuItem("Analyze Selected
Images");
    private JMenuItem filemenuItem = new JMenuItem("Quit");
    private JMenuItem settingsmenuItem2 = new JMenuItem("Export Settings
File");
    private JMenuBar menuBar = new JMenuBar();

    private Input_Settings_Panel settingsinfo = new Input_Settings_Panel();



    static public JButton processButton;

    public JMenuBar createMenuBar2() {
        ///////////////////////////////
        //MENU-BAR SETUP:
        //Set up the lone menu.
        JMenu filem = new JMenu("File");
        filem.setMnemonic(KeyEvent.VK_F);
        menuBar.add(filem);
        //
        JMenu settingsm = new JMenu("Settings");
        settingsm.setMnemonic(KeyEvent.VK_S);
        menuBar.add(settingsm);
        //
        JMenu analyzem = new JMenu("Analyze");
        analyzem.setMnemonic(KeyEvent.VK_A);
```

```
menuBar.add(analyzem);
//
JMenu helpm = new JMenu("Help");
helpm.setMnemonic(KeyEvent.VK_H);
menuBar.add(helpm);
///////////////////////////////
////Set up the FILE menu items.
filemenuItem.setMnemonic(KeyEvent.VK_Q);
filemenuItem.setAccelerator(KeyStroke.getKeyStroke(
        KeyEvent.VK_Q, ActionEvent.ALT_MASK));
filemenuItem.setActionCommand("quit");
filemenuItem.addActionListener(new menutButtonPress());
filem.add(filemenuItem);

////Set up the Settings menu items.
settingsmenuItem.setMnemonic(KeyEvent.VK_I);
settingsmenuItem.setAccelerator(KeyStroke.getKeyStroke(
        KeyEvent.VK_I, ActionEvent.ALT_MASK));
settingsmenuItem.setActionCommand("import_settings");
settingsmenuItem.addActionListener(new menutButtonPress());
settingsm.add(settingsmenuItem);

//Set up the second menu item.

settingsmenuItem2.setMnemonic(KeyEvent.VK_X);
settingsmenuItem2.setAccelerator(KeyStroke.getKeyStroke(
        KeyEvent.VK_X, ActionEvent.ALT_MASK));
settingsmenuItem2.setActionCommand("export_settings");
settingsmenuItem2.addActionListener(new menutButtonPress());
settingsm.add(settingsmenuItem2);

///////////////////////////////
////Set up the Analyze menu items.
analyzesmenuItem.setMnemonic(KeyEvent.VK_A);
analyzesmenuItem.setAccelerator(KeyStroke.getKeyStroke(
        KeyEvent.VK_A, ActionEvent.ALT_MASK));
analyzesmenuItem.setActionCommand("analyze_images");
analyzesmenuItem.addActionListener(new menutButtonPress());
analyzem.add(analyzesmenuItem);
///////////////////////////////
////Set up the Help menu items.
JMenuItem helpmenuitem = new JMenuItem("See online help");
helpmenuitem.setMnemonic(KeyEvent.VK_H);
helpmenuitem.setAccelerator(KeyStroke.getKeyStroke(
        KeyEvent.VK_H, ActionEvent.ALT_MASK));
helpmenuitem.setActionCommand("help_menu");
```

```
        helpmenuitem.addActionListener(new menutButtonPress());
        helpm.add(helpmenuitem);
        return menuBar;
    }

     private class menutButtonPress implements ActionListener {
    @Override
     public void actionPerformed(ActionEvent e){

        if ("set_change".equals(e.getActionCommand())) {
        } else if("quit".equals(e.getActionCommand())) { //quit
            quit();
        } else if("import_settings".equals(e.getActionCommand())) { //quit
        settingsinfo.getloadbutton().doClick();




        } else if("analyze_images".equals(e.getActionCommand())) { //quit



        if(processButton != null){
        //System.out.println("button is ON");
           processButton.doClick();
        }


        } else if("edit_settings".equals(e.getActionCommand())) { //quit



        } else if("export_settings".equals(e.getActionCommand())) { //quit
         settingsinfo.getsavebutton().doClick();

        }

     }
}


     public JMenuItem getAnalyzeButton(){
        return analyzesmenuItem;
     }

     public JMenuItem getImportButton(){
```

```java
        return settingsmenuItem;
    }

    public JMenuItem getExportButton(){
        return settingsmenuItem2;
    }

    public void placedir(JButton button){
       processButton = button;

   }

   protected void quit() {
       System.exit(0);
   }



}
```
