# Supplementary material for: Satellite cell heterogeneity revealed by G-Tool, an open algorithm to quantify myogenesis through colony-forming assays
Source: Skelet Muscle. 2012 Jun 15;2:13. doi: 10.1186/2044-5040-2-13 (PMC3439689; doi:10.1186/2044-5040-2-13)
Supplement: Additional file 1 — G-Tool Source Code. Java and MATLAB Source Codes are included. [file 2044-5040-2-13-S1.zip › G-Tool Sourcecode and PDF files/PDF files of code/JAVA - GUI/Output_Settings_Panel.pdf]

```
/*%     This file is part of GTOOL. AUTHOR: JOSEPH IPPOLITO, THE UNIVERSITY
%       OF MINNESOTA. GTOOL is free software: you can redistribute it
%   and/or modify
%       it under the terms of the GNU General Public License as published
%   by the Free Software Foundation, either version 3 of the License, or
%       (at your option) any later version.
%       GTOOL is distributed in the hope that it will be useful,
%   but WITHOUT ANY WARRANTY; without even the implied warranty of
%   MERCHANTABILITY or FITNESS FOR A PARTICULAR PURPOSE. SEE THE GNU
%   GENERAL PUBLIC LISCENCE FOR MORE DETAILS.
%       You should have received a copy of the GNU General Public License
%   along with GTOOL. If not see see <http://www.gnu.org/licenses/>. */
package gtool;
import java.util.*;
import java.awt.event.*;
import java.awt.*;
import javax.swing.*;
public class Output_Settings_Panel extends JPanel {

    private JPanel buttonpanel1 = new JPanel(new GridLayout(25,1));

    private JRadioButton save_trace_files_ON = new JRadioButton("Save Trace
Images");
    private JRadioButton save_trace_files_OFF = new JRadioButton("Don't
Save Trace Images");
    private int save_trace_counter;

    private JRadioButton process_options_SILENT = new JRadioButton("Silent
(no image displayed)");
    private JRadioButton process_options_NORMAL = new JRadioButton
("Normal");
    private int process_options_counter;

    private JRadioButton data_options_savecontinuously = new JRadioButton
("Continuously");
    private JRadioButton data_options_dontsave = new JRadioButton("Don't
Save");
    private int data_options_counter;

    private JRadioButton Nuclear_Stain_D = new JRadioButton("DAPI/BLUE");
    private JRadioButton Nuclear_Stain_R = new JRadioButton("RED");
    private JRadioButton Nuclear_Stain_G = new JRadioButton("GREEN");
    private int Nuclear_Stain_counter;

    private JRadioButton Primary_Stain_R = new JRadioButton("RED");
    private JRadioButton Primary_Stain_G = new JRadioButton("GREEN");
```

```
    private JRadioButton Primary_Stain_B = new JRadioButton("BLUE");
    private JRadioButton Primary_Stain_N = new JRadioButton("NONE");
    private int Primary_Stain_counter;

    private JRadioButton Secondary_Stain_R = new JRadioButton("RED");
    private JRadioButton Secondary_Stain_G = new JRadioButton("GREEN");
    private JRadioButton Secondary_Stain_B = new JRadioButton("BLUE");
    private JRadioButton Secondary_Stain_N = new JRadioButton("NONE");
    private int Secondary_Stain_counter;

    private JRadioButton counting_options_Fusions_OFF = new JRadioButton
("No Cell Fusions");
    private JRadioButton counting_options_Fusions_ON = new JRadioButton
("Consider Fusions");
    private int counting_options_counter;


       public Output_Settings_Panel() {

        //
        save_trace_files_ON.setActionCommand("1");
        save_trace_files_ON.addActionListener(new
Settings_radiobutton_actionlistener());
        save_trace_files_OFF.setActionCommand("2");
        save_trace_files_OFF.addActionListener(new
Settings_radiobutton_actionlistener());
        //
        process_options_SILENT.setActionCommand("3");
        process_options_SILENT.addActionListener(new
Settings_radiobutton_actionlistener());
        process_options_NORMAL.setActionCommand("4");
        process_options_NORMAL.addActionListener(new
Settings_radiobutton_actionlistener());
        //
        data_options_savecontinuously.setActionCommand("6");
        data_options_savecontinuously.addActionListener(new
Settings_radiobutton_actionlistener());
        data_options_dontsave.setActionCommand("7");
        data_options_dontsave.addActionListener(new
Settings_radiobutton_actionlistener());
        //
        Nuclear_Stain_D.setActionCommand("8");
        Nuclear_Stain_D.addActionListener(new
Settings_radiobutton_actionlistener());
        Nuclear_Stain_R.setActionCommand("9");
        Nuclear_Stain_R.addActionListener(new
```

```
Settings_radiobutton_actionlistener());
        Nuclear_Stain_G.setActionCommand("10");
        Nuclear_Stain_G.addActionListener(new
Settings_radiobutton_actionlistener());
        //
        Primary_Stain_R.setActionCommand("12");
        Primary_Stain_R.addActionListener(new
Settings_radiobutton_actionlistener());
        Primary_Stain_G.setActionCommand("13");
        Primary_Stain_G.addActionListener(new
Settings_radiobutton_actionlistener());
        Primary_Stain_B.setActionCommand("14");
        Primary_Stain_B.addActionListener(new
Settings_radiobutton_actionlistener());
        Primary_Stain_N.setActionCommand("15");
        Primary_Stain_N.addActionListener(new
Settings_radiobutton_actionlistener());
        //
        Secondary_Stain_R.setActionCommand("16");
        Secondary_Stain_R.addActionListener(new
Settings_radiobutton_actionlistener());
        Secondary_Stain_G.setActionCommand("17");
        Secondary_Stain_G.addActionListener(new
Settings_radiobutton_actionlistener());
        Secondary_Stain_B.setActionCommand("18");
        Secondary_Stain_B.addActionListener(new
Settings_radiobutton_actionlistener());
        Secondary_Stain_N.setActionCommand("19");
        Secondary_Stain_N.addActionListener(new
Settings_radiobutton_actionlistener());
        //
        counting_options_Fusions_ON.setActionCommand("20");
        counting_options_Fusions_ON.addActionListener(new
Settings_radiobutton_actionlistener());
        counting_options_Fusions_OFF.setActionCommand("21");
        counting_options_Fusions_OFF.addActionListener(new
Settings_radiobutton_actionlistener());



        ButtonGroup save_trace_group = new ButtonGroup();
        ButtonGroup process_options_group = new ButtonGroup();
        ButtonGroup data_options_group = new ButtonGroup();
        ButtonGroup Nuclear_Stain_Options = new ButtonGroup();
        ButtonGroup Primary_Stain_Options = new ButtonGroup();
        ButtonGroup Secondary_Stain_Options = new ButtonGroup();
```

```
        ButtonGroup fusion_group = new ButtonGroup();

        save_trace_group.add(save_trace_files_ON);
        save_trace_group.add(save_trace_files_OFF);
        process_options_group.add(process_options_SILENT);
        process_options_group.add(process_options_NORMAL);
        data_options_group.add(data_options_savecontinuously);
        data_options_group.add(data_options_dontsave);


         Nuclear_Stain_Options.add(Nuclear_Stain_D);
         Nuclear_Stain_Options.add(Nuclear_Stain_R);
         Nuclear_Stain_Options.add(Nuclear_Stain_G);

         Primary_Stain_Options.add(Primary_Stain_R);
         Primary_Stain_Options.add(Primary_Stain_G);
         Primary_Stain_Options.add(Primary_Stain_B);
         Primary_Stain_Options.add(Primary_Stain_N);

         Secondary_Stain_Options.add(Secondary_Stain_R);
         Secondary_Stain_Options.add(Secondary_Stain_G);
         Secondary_Stain_Options.add(Secondary_Stain_B);
         Secondary_Stain_Options.add(Secondary_Stain_N);
         fusion_group.add(counting_options_Fusions_ON);
         fusion_group.add(counting_options_Fusions_OFF);


        JPanel TRACE_BUTTONGROUP_PANEL = new JPanel(new GridLayout(1, 3));
        JPanel PROCESS_BUTTONGROUP_PANEL = new JPanel(new GridLayout(1,
3));
        JPanel DATA_BUTTONGROUP_PANEL = new JPanel(new GridLayout(1, 3));
        JPanel Nuclear_Stain_Panel = new JPanel(new GridLayout(1,4));
        JPanel Primary_Stain_Panel = new JPanel(new GridLayout(1,4));
        JPanel Secondary_Stain_Panel = new JPanel(new GridLayout(1,3));



        JPanel fusion_setting_panel = new JPanel(new GridLayout(1,3));

        TRACE_BUTTONGROUP_PANEL.add(save_trace_files_ON);
        TRACE_BUTTONGROUP_PANEL.add(save_trace_files_OFF);
        TRACE_BUTTONGROUP_PANEL.add(new JLabel(""));

        PROCESS_BUTTONGROUP_PANEL.add(process_options_SILENT);
        PROCESS_BUTTONGROUP_PANEL.add(process_options_NORMAL);
        PROCESS_BUTTONGROUP_PANEL.add(new JLabel(""));
```

```
DATA_BUTTONGROUP_PANEL.add(data_options_savecontinuously);
DATA_BUTTONGROUP_PANEL.add(data_options_dontsave);
DATA_BUTTONGROUP_PANEL.add(new JLabel(""));

 Nuclear_Stain_D.setForeground(Color.blue);
 Nuclear_Stain_Panel.add(Nuclear_Stain_D);

 Nuclear_Stain_Panel.add(Nuclear_Stain_R);
 Nuclear_Stain_R.setForeground(Color.red);

 Nuclear_Stain_Panel.add(Nuclear_Stain_G);
 Nuclear_Stain_G.setForeground(new Color(34,139,34));
 Nuclear_Stain_Panel.add(new JLabel(""));


 Primary_Stain_R.setForeground(Color.red);
 Primary_Stain_Panel.add(Primary_Stain_R);

 Primary_Stain_G.setForeground(new Color(34,139,34));
 Primary_Stain_Panel.add(Primary_Stain_G);

 Primary_Stain_B.setForeground(Color.blue);
 Primary_Stain_Panel.add(Primary_Stain_B);

 Primary_Stain_Panel.add(Primary_Stain_N);

 Secondary_Stain_R.setForeground(Color.red);
 Secondary_Stain_Panel.add(Secondary_Stain_R);

 Secondary_Stain_Panel.add(Secondary_Stain_G);
 Secondary_Stain_G.setForeground(new Color(34,139,34));

 Secondary_Stain_Panel.add(Secondary_Stain_B);
 Secondary_Stain_B.setForeground(Color.blue);

 Secondary_Stain_Panel.add(Secondary_Stain_N);

//fusion_setting_panel.add(counting_options_Fusions_ON);
//fusion_setting_panel.add(counting_options_Fusions_OFF);
//fusion_setting_panel.add(new JLabel(""));
//DEFAULT
save_trace_files_ON.doClick();
process_options_NORMAL.doClick();
data_options_savecontinuously.doClick();
Nuclear_Stain_D.doClick();
```

```
        Primary_Stain_R.doClick();
        Secondary_Stain_N.doClick();

        counting_options_Fusions_ON.doClick();

        JLabel trace_settings_label = new JLabel("Trace Images (ROI) Output
Settings:");
        JLabel process_settings_label = new JLabel("Image Processing/
Display Options:");
        JLabel data_settings_label = new JLabel("Data Settings: Save
Data");

        buttonpanel1.add(new JLabel(""));
        buttonpanel1.add(new JLabel(""));


        buttonpanel1.add(trace_settings_label);
        buttonpanel1.add(TRACE_BUTTONGROUP_PANEL);
        buttonpanel1.add(new JSeparator());
        buttonpanel1.add(new JSeparator());


//        buttonpanel1.add(process_settings_label);
//        buttonpanel1.add(PROCESS_BUTTONGROUP_PANEL);
//        buttonpanel1.add(new JSeparator());
//        buttonpanel1.add(new JSeparator());


        buttonpanel1.add(data_settings_label);
        buttonpanel1.add(DATA_BUTTONGROUP_PANEL);
        buttonpanel1.add(new JSeparator());
        buttonpanel1.add(new JSeparator());
        //9

        buttonpanel1.add(new JLabel("Nuclear Stain: "));
        buttonpanel1.add(Nuclear_Stain_Panel);
        buttonpanel1.add(new JSeparator());
        buttonpanel1.add(new JSeparator());

        buttonpanel1.add(new JLabel("Primary Stain: "));
        buttonpanel1.add(Primary_Stain_Panel);
        buttonpanel1.add(new JSeparator());
        buttonpanel1.add(new JSeparator());

        buttonpanel1.add(new JLabel("Secondary Stain: "));
        buttonpanel1.add(Secondary_Stain_Panel);
```

```
         buttonpanel1.add(new JSeparator());
         buttonpanel1.add(new JSeparator());


        // buttonpanel1.add(new JLabel("ADVANCED SETTINGS:"));
        // buttonpanel1.add(fusion_setting_panel);
        // buttonpanel1.add(new JSeparator());
        // buttonpanel1.add(new JSeparator());

         this.add(buttonpanel1);

}


        public ArrayList getSettings(){
         ArrayList SettingsList = new ArrayList();

         SettingsList.add(save_trace_counter);
         SettingsList.add(process_options_counter);
         SettingsList.add(data_options_counter);
         SettingsList.add(Nuclear_Stain_counter);
         SettingsList.add(Primary_Stain_counter);
         SettingsList.add(Secondary_Stain_counter);
         SettingsList.add(counting_options_counter);


         return SettingsList;
        }




        protected class Settings_radiobutton_actionlistener implements
ActionListener {
         public void actionPerformed(ActionEvent e) {


          String actionC = e.getActionCommand();
          int casex = Integer.parseInt(actionC);
                          //System.out.println(casex);
                          //System.out.println(actionC);


          switch (casex){
              case 1:
                  System.out.println("Save Trace Files ON");
```

```
        save_trace_counter = 1;
        break;
    case 2:
        System.out.println("Save Trace Files OFF");
        save_trace_counter = 0;
        break;
    case 3:
        System.out.println("Rigged for SILENT RUNNING");
        process_options_counter = 0;
        break;
    case 4:
        System.out.println("Rigged for NORMAL RUNNING");
        process_options_counter = 1;
        break;
    case 6:
        System.out.println("Save DATA Continuously");
        data_options_counter = 1;
        break;
    case 7:
        System.out.println("Don't Save DATA");
        data_options_counter = 2;
        break;
    case 8:
        System.out.println("Nuclear Stain = DAPI");
        Nuclear_Stain_counter = 1;
        break;
    case 9:
        System.out.println("Nuclear Stain = Red");
        Nuclear_Stain_counter = 2;
        break;
    case 10:
        System.out.println("Nuclear Stain = Green");
        Nuclear_Stain_counter = 3;
        break;
    case 12:
        System.out.println("Primary Stain = Red");
        Primary_Stain_counter = 1;
        break;
    case 13:
        System.out.println("Primary Stain = Green");
        Primary_Stain_counter = 2;
        break;
    case 14:
        System.out.println("Primary Stain = Blue");
        Primary_Stain_counter = 3;
        break;
```

```
            case 15:
                System.out.println("Primary Stain = NONE!!");
                Primary_Stain_counter = 4;
                break;
            case 16:
                System.out.println("Secondary Stain = Red");
                Secondary_Stain_counter = 1;
                break;
            case 17:
                System.out.println("Secondary Stain = Green");
                Secondary_Stain_counter = 2;
                break;
            case 18:
                System.out.println("Secondary Stain = Blue");
                Secondary_Stain_counter = 3;
                break;
            case 19:
                System.out.println("Secondary Stain = None!");
                Secondary_Stain_counter = 4;
                break;
            case 20:
                System.out.println("Counting Options: Fusions ON");
                counting_options_counter = 1;
                break;
            case 21:
                System.out.println("Counting Options: Fusions OFF");
                counting_options_counter = 0;
                break;
        }

} }
   }
```
