# Supplementary material for: Satellite cell heterogeneity revealed by G-Tool, an open algorithm to quantify myogenesis through colony-forming assays
Source: Skelet Muscle. 2012 Jun 15;2:13. doi: 10.1186/2044-5040-2-13 (PMC3439689; doi:10.1186/2044-5040-2-13)
Supplement: Additional file 1 — G-Tool Source Code. Java and MATLAB Source Codes are included. [file 2044-5040-2-13-S1.zip › G-Tool Sourcecode and PDF files/PDF files of code/JAVA - GUI/Settings_File_Loader.pdf]

```
/*%     This file is part of GTOOL. AUTHOR: JOSEPH IPPOLITO, THE UNIVERSITY
%       OF MINNESOTA. GTOOL is free software: you can redistribute it
%   and/or modify
%       it under the terms of the GNU General Public License as published
%   by the Free Software Foundation, either version 3 of the License, or
%       (at your option) any later version.
%       GTOOL is distributed in the hope that it will be useful,
%   but WITHOUT ANY WARRANTY; without even the implied warranty of
%   MERCHANTABILITY or FITNESS FOR A PARTICULAR PURPOSE. SEE THE GNU
%   GENERAL PUBLIC LISCENCE FOR MORE DETAILS.
%       You should have received a copy of the GNU General Public License
%   along with GTOOL. If not see see <http://www.gnu.org/licenses/>. */
package gtool;
import java.io.*;
import javax.swing.*;
import java.util.*;
public class Settings_File_Loader extends JPanel {
    double i;
    double d;
    boolean b;
    String str;
    double j = 0;
    double[] doublevariables;
    // Main method
        public void savefile (String args,double DAPI_CONTRAST, double
RED_CONTRAST, double GREEN_CONTRAST,double DAPI_SENSITIVITY,
                double RED_SENSITIVITY,double GREEN_SENSITIVITY,double
DAPI_BLUR_SPINNER, double RED_BLUR_SPINNER,double GREEN_BLUR_SPINNER,double
fpc, double fpl, double fpu,double
dapi_auto_or_manual_contrast_counter,double
red_auto_or_manual_contrast_counter,double
green_auto_or_manual_contrast_counter,
                double dapi_auto_or_manual_sensitivity_counter,double
red_auto_or_manual_sensitivity_counter,double
green_auto_or_manual_sensitivity_counter)
        {

            // Stream to write file
                FileOutputStream fout;
                System.out.println("In load file 1");

                try
                {
                    // Open an output stream
                    fout = new FileOutputStream (args);
```

```
                    System.out.println("Writing file..");
                    System.out.println("DAPI_CONTRAST = " + DAPI_CONTRAST);
                    System.out.println("DAPI_SENSITIVITY = " +
DAPI_SENSITIVITY);

                    // Prdouble a line of text
                   new PrintStream(fout).println (DAPI_CONTRAST + "    =
DAPI Contrast slider value");
                   new PrintStream(fout).println (RED_CONTRAST + "    = RED
Contrast slider value");
                   new PrintStream(fout).println (GREEN_CONTRAST + "    =
GREEN Contrast slider value");

                   new PrintStream(fout).println (DAPI_SENSITIVITY + "    =
DAPI Sensitivity slider value");
                   new PrintStream(fout).println (RED_SENSITIVITY + "    =
RED Sensitivity slider value");
                   new PrintStream(fout).println (GREEN_SENSITIVITY + "
= GREEN Sensitivity slider value");

                   new PrintStream(fout).println (DAPI_BLUR_SPINNER + "
= DAPI Blur spinner value");
                   new PrintStream(fout).println (RED_BLUR_SPINNER + "    =
RED Blur spinner value");
                   new PrintStream(fout).println (GREEN_BLUR_SPINNER + "
= GREEN Blur spinner value");

                   new PrintStream(fout).println (fpc + "    = First peak
center");
                   new PrintStream(fout).println (fpl + "    = First peak
lower bound");
                   new PrintStream(fout).println (fpu + "    = First peak
upper bound");

                   new PrintStream(fout).println
(dapi_auto_or_manual_contrast_counter + "    = DAPI contrast auto or
manual");
                   new PrintStream(fout).println
(red_auto_or_manual_contrast_counter + "    = RED contrast auto or
manual");
                   new PrintStream(fout).println
(green_auto_or_manual_contrast_counter + "    = GREEN contrast auto or
manual");

                   new PrintStream(fout).println
(dapi_auto_or_manual_sensitivity_counter + "    = DAPI sensitivity auto or
```

```
manual");
                   new PrintStream(fout).println
(red_auto_or_manual_sensitivity_counter + "    = RED sensitivity auto or
manual");
                   new PrintStream(fout).println
(green_auto_or_manual_sensitivity_counter + "    = GREEN sensitivity auto
or manual");

                    System.out.println("File written, are there errors?");


                    // Close our output stream
                    fout.close();
                }
                // Catches any error conditions
                catch (IOException e)
                {
                        System.err.println ("Unable to write to file");
                        System.exit(-1);
                }
        }


        public double[] loadfile (String args){
            doublevariables = new double[18];

            try{

         FileReader fin = new FileReader(args);

         Scanner src = new Scanner(fin);
          while (src.hasNext()) {
            if (src.hasNextDouble()) {
         i = src.nextDouble();
        //System.out.println("double: " + i);
        doublevariables[(int)j] = i;
        //System.out.println("doublevariable " + doublevariables[(int)j]);
        j++;

      } else if (src.hasNextDouble()) {
        d = src.nextDouble();
        //System.out.println("double: " + d);
      } else if (src.hasNextBoolean()) {
        b = src.nextBoolean();
        //System.out.println("boolean: " + b);
      } else {
```

```
            str = src.next();
            //System.out.println("String: " + str);
          }
        }

              fin.close();

            }catch (Exception e){//Catch exception if any
                     System.err.println("Error: " + e.getMessage());
                     }

                     return doublevariables;


            }


    }
```
