# Supplementary material for: Satellite cell heterogeneity revealed by G-Tool, an open algorithm to quantify myogenesis through colony-forming assays
Source: Skelet Muscle. 2012 Jun 15;2:13. doi: 10.1186/2044-5040-2-13 (PMC3439689; doi:10.1186/2044-5040-2-13)
Supplement: Additional file 1 — G-Tool Source Code. Java and MATLAB Source Codes are included. [file 2044-5040-2-13-S1.zip › G-Tool Sourcecode and PDF files/PDF files of code/MATLAB - Algorithm/calibration.pdf]

```
function [areaA] = calibration(image_list,blur_factor,disk1,contrast_threshold1)
%   This file is part of GTOOL. AUTHOR: JOSEPH IPPOLITO, THE UNIVERSITY
%   OF MINNESOTA. GTOOL is free software: you can redistribute it
%   and/or modify
%   it under the terms of the GNU General Public License as published
%   by the Free Software Foundation, either version 3 of the License, or
%   (at your option) any later version.
%   GTOOL is distributed in the hope that it will be useful,
%   but WITHOUT ANY WARRANTY; without even the implied warranty of
%   MERCHANTABILITY or FITNESS FOR A PARTICULAR PURPOSE. SEE THE GNU
%   GENERAL PUBLIC LISCENCE FOR MORE DETAILS.
%   You should have received a copy of the GNU General Public License
%   along with GTOOL. If not see see <http://www.gnu.org/licenses/>.
debug = 1;

if debug == 1
    clear; clc; close all
        %Edit the following for quick algorithm testing:
    debug = 1;
    blur_factor = 1;
    disk1 = 5;  contrast_threshold1 = 10;
    addpath('C:\Documents and Settings\Jack\Desktop\GTOOL Java\M Files')
end
 warning off all; display('Calibration Program Running...');
 if debug == 1
    %Edit the following for quick algorithm testing:
     dir1 = 'C:\Muscle Group Experiment Images\9871\9871 Tricep';
     cd(dir1)
     image_list = dir('9871*.JPG'); %select only images that start with 9871, edit this.
     image_array = cell(length(image_list),1);
    for i = 1:length(image_list)
        image_array{i,1} = image_list(i).name;
    end
 end
    [number_of_images,cee] = size(image_list);
    area = cell(1,number_of_images);
for image_counter = 1:number_of_images
    display(['Processing Image Number ', num2str(image_counter),' of ',num2str(number_of_images)]);
    if debug == 1
        filename1 = image_array{image_counter,1};
    else
        filename1 = image_list(image_counter,:);
    end
    Ig = uint8(imread(filename1));
    Gaussian_filter = fspecial('gaussian',[5 5],10);
    if blur_factor >= 1
        for i = 1:blur_factor
            IgD = imfilter(Ig,Gaussian_filter,'same');
        end
    else
        IgD = Ig;
    end
    Image_stage2      = IgD(:,:,3) - imopen(IgD(:,:,3),strel('disk',round(double(disk1))));
    Image_stage2(Image_stage2 >= contrast_threshold1) = 255;%Anything above threshold value is now totally↙
blue
    Image_stage2(Image_stage2 <  contrast_threshold1)  = 0;%Anything below threshold value is now totally↙
black
    [Bd]     = bwboundaries(Image_stage2,4,'noholes');
    LBD      = length(Bd);
    clear centroidBlue x_sum image_names
    x_sum = zeros(1,LBD);
    for k=1:LBD
       boundary = Bd{k};
       bx = boundary(:,1);
       by = boundary(:,2);
    try
       if length(bx)> 2 && length(by) > 2 && length(find(bx == mean(bx))) ~= length(bx) && length(find(by↙
== mean(by))) ~= length(by)
            y = polygeom(bx,by);
            x_sum(k) = y(1); %Area
       end
    end
    end
    area{:,image_counter} = x_sum';
end
```

```
   try
    if number_of_images ~= 1
        areaA       = catpad(1,area{1:length(area)});
        areaA(areaA == 0) = [];
    else
        areaA = area{1:length(area)};
    end
    figure('units','normalized','outerposition',[0 0 1 1])
    hist(areaA(areaA<2*mean(areaA)),25);
    display(['Mean of region areas = ', num2str(mean(areaA))]);
    display(['Mode of region areas = ', num2str(mode(areaA))]);
    display(['St.dev of region areas = ', num2str(std(areaA))]);
    display(['Number of regions scanned = ', num2str(length(areaA))]);
    set(get(gca,'child'),'FaceColor','none','EdgeColor','k','LineWidth',3);
    hTitle  = title ('Histogram of DAPI Region Sizes');
    hXLabel = xlabel('Algorithm Determined Region Sizes in Pixels');
    hYLabel = ylabel('Counts');
    set( gca,'FontName', 'Helvetica' );
    set([hTitle, hXLabel, hYLabel],'FontName', 'AvantGarde');
    set([hXLabel,hYLabel],'FontSize', 10);
    set( hTitle,'FontSize', 12,'FontWeight', 'bold');
    set(gca,'Box', 'off','TickDir','out','TickLength',[.02 .02],'XMinorTick', 'on','YMinorTick',↙
'on','YGrid', 'on','XColor', [.3 .3 .3],'YColor', [.3 .3 .3],'XTick', 1:5:2*mean(areaA),'LineWidth', 1);
   catch
       display('Histogram plotting failure. Uknown Error has occured')
   end
display('Calibration program finished. Please look at the Histogram and select the appropriate nuclear↙
settings');
```
