# Supplementary material for: Satellite cell heterogeneity revealed by G-Tool, an open algorithm to quantify myogenesis through colony-forming assays
Source: Skelet Muscle. 2012 Jun 15;2:13. doi: 10.1186/2044-5040-2-13 (PMC3439689; doi:10.1186/2044-5040-2-13)
Supplement: Additional file 1 — G-Tool Source Code. Java and MATLAB Source Codes are included. [file 2044-5040-2-13-S1.zip › G-Tool Sourcecode and PDF files/PDF files of code/MATLAB - Algorithm/catpad.pdf]

```
function [M, TF] = catpad(dim,varargin)
% Copyright (c) 2011, Jonathan Sullivan
% Copyright (c) 2011, Michael Völker
% All rights reserved.
%
% Redistribution and use in source and binary forms, with or without
% modification, are permitted provided that the following conditions are
% met:
%
%     * Redistributions of source code must retain the above copyright
%       notice, this list of conditions and the following disclaimer.
%     * Redistributions in binary form must reproduce the above copyright
%       notice, this list of conditions and the following disclaimer in
%       the documentation and/or other materials provided with the distribution
%
% THIS SOFTWARE IS PROVIDED BY THE COPYRIGHT HOLDERS AND CONTRIBUTORS "AS IS"
% AND ANY EXPRESS OR IMPLIED WARRANTIES, INCLUDING, BUT NOT LIMITED TO, THE
% IMPLIED WARRANTIES OF MERCHANTABILITY AND FITNESS FOR A PARTICULAR PURPOSE
% ARE DISCLAIMED. IN NO EVENT SHALL THE COPYRIGHT OWNER OR CONTRIBUTORS BE
% LIABLE FOR ANY DIRECT, INDIRECT, INCIDENTAL, SPECIAL, EXEMPLARY, OR
% CONSEQUENTIAL DAMAGES (INCLUDING, BUT NOT LIMITED TO, PROCUREMENT OF
% SUBSTITUTE GOODS OR SERVICES; LOSS OF USE, DATA, OR PROFITS; OR BUSINESS
% INTERRUPTION) HOWEVER CAUSED AND ON ANY THEORY OF LIABILITY, WHETHER IN
% CONTRACT, STRICT LIABILITY, OR TORT (INCLUDING NEGLIGENCE OR OTHERWISE)
% ARISING IN ANY WAY OUT OF THE USE OF THIS SOFTWARE, EVEN IF ADVISED OF THE
% POSSIBILITY OF SUCH DAMAGE.

% CATPAD - concatenate arrays with any size by padding with a given value.
%
%   M = CATPAD(dim,A1, A2, A3, ..., AN) concatenates along the dimension
%       DIM the arrays A1 through AN into one large matrix. The vectors do
%       not need to have the same size, nor number of dimensions. The size
%       of the output M is determined by the  dimmention of concatenation,
%       and the size of the inputs. Any inputs that are not the correct
%       size will be padded with NaNs if the inputs are numeric. If they
%       are strings, they will be padded with a space " ".
%
%   [M TF] = CATPAD(...,'padval',padval) pads the input data with the value
%       specified by PADVAL. The default is NaN for numeric inputs, and a
%       space " " for string inputs.
%
%   [M TF] = CATPAD(...) outputs ana array of logicals TF having true
%       values when the values in that position of M were from the original
%       data (i.e. not padded).
%
%   Examples:
%      a = 1:4; b = 1:5; c = []; d = 1:3; dim = 1;
%      M = catpad(dim,a,b,c,d)
%      M =
%          1     2     3     4   NaN
%          1     2     3     4     5
%          1     2     3   NaN   NaN
%      % Note: The input "c" was empty, and therefore doesn't require it's
%           % own row.
%
%      a = rand(3); b = magic(5); c = rand([4 5 4]); dim = 3;
%      M = catpad(dim,a,b,c);
%      size(M)
%           5     5     6
%
%      str1 = 'What do you think of this function?';
%      str2 = 'I like it!';
%      dim = 1;
%      M = catpad(dim,str1,str2)
%      M =
%           What do you think of this function?
%           I like it!
%
%   Example: Find original NaNs
%       a = 1:3; b = [2:5 NaN]; c = [1 NaN]; dim = 1;
%       [M,tf] = catpad(dim,a,b,c)
%       % find the original NaN
%       [row,col] = find(tf & isnan(M))
%       % -> row = [3 2] , col = [2 5]
%
```

```
%       a = 1:3; b = 1; c = 1:4; dim = 1; padval = inf;
%       M = catpad(dim,a,b,c,'padval',padval)
%       M =
%              1     2     3   Inf
%              1   Inf   Inf   Inf
%              1     2     3     4
%
%   See also CAT, RESHAPE, STRVCAT, CHAR, HORZCAT, VERTCAT, ISEMPTY
%
% By Jonathan Sullian - October 2011
%
% Many thanks to Michael Völker for his function sub2allind which has made
%   this function possible. Also, I would like to aknowledge Jos (10584)
%   whose submission padcat has inspired this one.

% Error Checking
if numel(dim) ~= 1
    error('catpad:DimSize', 'The input DIM must be a scalar.')
elseif ~(dim >= 0  &&  dim==round(dim))
    error('catpad:DimValue','The input DIM must be a positive interger.');
end

% Extract the pad value (if specified).
if strcmpi(varargin{end-1},'padval')
    pval = varargin{end};
    shouldBeCells = cellfun(@iscell,varargin(1:end-2));
    if ~any(shouldBeCells);
        if numel(pval) ~= 1
            error('catpad:PadValueSize', 'The value of PADVAL must be a scalar.')
        elseif iscell(pval)
            error('catpad:PadValueType', 'PADVAL cannot be a cell if none of the inputs are cells.')
        end
    end
    varargin = varargin(1:end-2);
else
    pval = NaN;
    shouldBeCells = cellfun(@iscell,varargin);
end
n2cat = length(varargin);

% Handle Cells
if any(shouldBeCells)
    for ii = find(~shouldBeCells(1:end-1))
        varargin(ii) = {varargin(ii)};
    end
end
shouldBeCells = any(shouldBeCells);

% check the inputs
SZ = cellfun(@size,varargin,'UniformOutput',false) ; % sizes
nel = cellfun(@numel,SZ) ; % number of dimensions
shouldBeChar = all(cellfun(@ischar,varargin));



% Concatenate the sizes
if all(nel == nel(1))
    SZ = cat(1,SZ{:}) ;
else
    SZ = catpad(1,SZ{:});
end

% Non-specified sizes are 1
SZ(isnan(SZ)) = 1;

% Calculate the size of the output
maxSZ = max(SZ) ;
ndsIn = length(maxSZ);
if dim > ndsIn
    szOut = ones(1,dim);
    szOut(1:ndsIn) = maxSZ;
    maxSZ = szOut;
    szOut(dim) = n2cat;
    SZ = cat(2,SZ,ones(n2cat,dim-ndsIn));
else
```

```
    szOut = maxSZ;
    szOut(dim) = sum(SZ(:,dim));
end

% Allocate Memory
if shouldBeCells
    M = cell(szOut);
else
    M = NaN(szOut);
end
TF = false(szOut);

% Place the input data
for ii = 1:n2cat

    % Skip any empty inputs.
    if isempty(varargin{ii}); continue; end

    % Get block size where the data is going to be placed.
    maxSZ(dim) = SZ(ii,dim);
    useMe = false(maxSZ);

    % Create logical indexing of where it is going to be place.
    um = arrayfun(@(in2) colon(1,in2),SZ(ii,:),'UniformOutput',false);
    useMe(sub2allind(maxSZ,um{:})) = true;
    rm1 = szOut;
    rm2 = szOut;
    if dim > ndsIn
        rm1(dim) = ii-1;
        rm2(dim) = n2cat-ii;
    else
        rm1(dim) = sum(SZ(1:(ii - 1),dim));
        rm2(dim) = sum(SZ((ii+1):end,dim));
    end
    ind = cat(dim,false(rm1),useMe,false(rm2));

    % Place the data and update the TF matrix.
    M(ind) = varargin{ii};
    TF = TF | ind;
end

% By convention
if isempty(TF);
    TF = true;
end

% Replace the padded value.
if shouldBeCells
    M(~TF) = {pval};
else
    M(~TF) = pval;
end

% Convert to strings.
if shouldBeChar
    if isnan(pval)
        M(~TF) = ' ';
    end
    M = char(M);
end

function idx = sub2allind(sz, varargin)
%   idx = sub2allind(sz, sub1, sub2, sub3, ... )
%
%   Like Matlab's sub2ind, sub2allind computes the equivalent linear indices for
%   given subscripts of an array with size SZ.
%   Unlike sub2ind, it computes a field of all combinations of
%   subscripts. So, instead of calling A( 2:3, 1, 4:11) you might
%   use
%       linIdx = sub2allind( size(A), 2:3, 1, 4:11 );
%
%   and then call A(linIdx) or A(linIdx(:)) or you reshape linIdx the way you need.
%
%   A(linIdx) has the shape of the cut out part of the array
%   A(linIdx(:)) is a column vector with all entries.
```

```
%
%   Example:
%   >> A = magic(4)
%
%   A =
%
%       16   _ 2 ___ 3 _  13
%        5  | 11    10  |  8
%        9  |  7     6  | 12
%        4  | 14    15  |  1
%
%   >> linIdx = sub2allind( size(A), 2:4, 2:3 );
%   >> A(linIdx)
%
%   ans =
%
%       11    10
%        7     6
%       14    15
%
%   >> A(linIdx(:)).'
%
%   ans =
%
%       11     7    14    10     6    15
%
%   Using the colon operator is allowed:
%   linIdx = sub2allind( sz, sub1, :, sub3 );

% Michael VÃ¶lker, 2011
% This is Matlab FileEx #30096

% ==================================================================
% Make sure, "sz" is fine
%
if nargin < 2
    error('sub2allind:NoOfInputs', 'At least two inputs, please.')
end
if ~exist('sz', 'var') || length(sz(:)) < 2 || ~all(isnumeric(sz(:))) || ~all(isfinite(sz(:))) || ~all↙
(isreal(sz(:)))
    error('sub2allind:szNotSane', 'Size vector must be real and have at least 2 elements.');
end
sz  = sz(:).';
Ndims = length(sz);

if Ndims < nargin-1
    ResDims = nargin-1 - Ndims;
    sz = [sz   ones(1,ResDims)];                     % Adjust for trailing singleton dimensions
elseif Ndims > nargin-1
    sz = [sz(1:nargin-2)  prod(sz(nargin-1:end))];  % Adjust for linear indexing on last element
end
% ==================================================================

Ndims = length(sz);     % No. of dimensions

% ==================================================================
% Make sure all subscripts are fine, including the use of ":"
%
for d = 1:Ndims
    subs = varargin{d}(:);
    % Check if:
    flagNum  = all( isnumeric( subs )         );      %   Subs are numeric...
    flagReal = all( isreal(    subs )         );      %   ... real valued ...
    flagFin  = all( isfinite(  subs )         );      %   ... finite ...
    flagInt  = all( subs == floor(subs)       );      %   ... integers.
    if ~flagNum || ~flagReal || ~flagFin || ~flagInt
        if isequal( subs, ':' )          % We allow exactly one type of non-numeric input
            varargin{d} = 1:sz(d);      % interpret ":" as in usual subscript syntax
        else
            error('sub2allind:SubsNotSane', 'Subscripts must either be valid matrix subscripts or the well↙
known '':''.')
        end

    end
    % subscripts within allowed range?
```

```
    if any( varargin{d} < 1  |  varargin{d} > sz(d) )
        error('sub2allind:IndexOutOfRange', 'Out of range subscript.');
    end
end
% ==================================================================


% ==================================================================
%  Compute linear indices, e.g. in 3D, with edges L1, L2, L3:
%
%       idx = x1 + (x2-1)*L1 + (x3-1)*L1*L2,
%
%  for every permutation (x1,x2,x3)
k = [1 cumprod(sz(1:end-1))];           % k(d) holds the accumulated No. of array elements
% up to the d'th subscript (or dimension)

idx = 1;                                % smallest possible index

for d = 1:Ndims
    xd = varargin{d}(:);                        % the d'th subscripts
    reshrule      = ones(1,d+1);                % how to reshape the size of xd
    reshrule(end) = length(xd);                 %
    xd = reshape( xd, reshrule );               % prepare for bsxfun's needs
    idx = bsxfun( @plus, idx, (xd-1)*k(d) );    % iteratively calculate the sum pointed out above

end
% ==================================================================

szIdx = size( idx );
if numel(szIdx) > 2
    idx = reshape( idx, szIdx(2:end) );
end
```
