# Supplementary material for: Satellite cell heterogeneity revealed by G-Tool, an open algorithm to quantify myogenesis through colony-forming assays
Source: Skelet Muscle. 2012 Jun 15;2:13. doi: 10.1186/2044-5040-2-13 (PMC3439689; doi:10.1186/2044-5040-2-13)
Supplement: Additional file 1 — G-Tool Source Code. Java and MATLAB Source Codes are included. [file 2044-5040-2-13-S1.zip › G-Tool Sourcecode and PDF files/PDF files of code/MATLAB - Algorithm/counting_program.pdf]

```
function [total_number_of_nuclei,stain_negative,single_positive,double_positive,triple_plus_positive,
fusion_index,negative_si,coeff_diff] = counting_program(stain_selection,image_directory,image_list,
blur_factor,blur_factorPrimary,blur_factorSecondary,disk1,disk2,disk3,contrast_threshold1,
contrast_threshold2,contrast_threshold3,first_peak_center,first_peak_lower,first_peak_upper,plot_or_not,
data_save,debug)
%   This file is part of GTOOL. AUTHOR: JOSEPH IPPOLITO, THE UNIVERSITY
%   OF MINNESOTA. GTOOL is free software: you can redistribute it
%   and/or modify
%   it under the terms of the GNU General Public License as published
%   by the Free Software Foundation, either version 3 of the License, or
%   (at your option) any later version.
%   GTOOL is distributed in the hope that it will be useful,
%   but WITHOUT ANY WARRANTY; without even the implied warranty of
%   MERCHANTABILITY or FITNESS FOR A PARTICULAR PURPOSE. SEE THE GNU
%   GENERAL PUBLIC LISCENCE FOR MORE DETAILS.
%   You should have received a copy of the GNU General Public License
%   along with GTOOL. If not see see <http://www.gnu.org/licenses/>.
format short g
debug = 0; %Edit to run the debug scripts in the code.
if debug == 1
    clear;clc;
    number_of_debug_images = 0;
    stain_selection = [1,1,4,1];
    debug = 1;
    blur_factor = 1; blur_factorPrimary = 1; blur_factorSecondary = 1;
    %%For quick algorithm testing (with debug=1) modify the following
    %%values:
     disk1 = 5;  contrast_threshold1 = 10; contrast_threshold3 = 30;
     disk2 = 63; contrast_threshold2 = 15; disk3 = 63;
     first_peak_center = 28; %first_peak_upper-first_peak_lower;
     first_peak_lower = 13/first_peak_center;
     first_peak_upper = 44/first_peak_center; plot_or_not = 1;data_save = 1;
else
    dir1 = image_directory;
    dir2 = dir1;
end
warning off all; display('Counting Program Active...');c = clock;
 if debug == 1
     %Debug image library location
     dir1 = 'C:\Muscle Group Experiment Images\9871\9871 Tricep\';
     dir2 = dir1;
     cd(dir1)
     image_list = dir('9871*.JPG'); %selects only images that start with 9871, edit this.
     image_array = cell(length(image_list),1);
    for i = 1:length(image_list)
        image_array{i,1} = image_list(i).name;
    end
 end
    [number_of_images,cee] = size(image_list);
    image_processing_k = zeros(1,number_of_images);
    area = cell(1,number_of_images);
    centroids = cell(1,number_of_images);
    image_name = cell(1,number_of_images);
    nuclear_list = cell(1,number_of_images);
    f_list = cell(1,number_of_images);
    single_positive      = zeros(1,number_of_images);
    double_positive      = zeros(1,number_of_images);
    triple_plus_positive = zeros(1,number_of_images);
    stain_negative       = zeros(1,number_of_images);
    total_number_of_nuclei = zeros(1,number_of_images);
    fusion_index         = zeros(1,number_of_images);
    negative_si          = zeros(1,number_of_images);
    coeff_diff           = zeros(1,number_of_images);

    folder_name = ['\Trace Images_' num2str(c(1)) '-' num2str(c(2)) '-' num2str(c(3)) '-' num2str(c(4))
'-' num2str(c(5)) '\'];
    dir3 = fullfile(dir2,folder_name);

    if plot_or_not ~= 0
        try
            trace_directory_success = mkdir(dir3);
        end
    end

    switch stain_selection(1) %Nuclear stain option
```

```
        case 1 %dapi selected
            nuclear_stain = 3;
        case 2 %red selected
            nuclear_stain = 1;
        case 3 %green selected
            nuclear_stain = 2;
    end

    switch stain_selection(2) %Primary stain option
        case 1 %dapi selected
            primary_stain = 1;
        case 2 %red selected
            primary_stain = 2;
        case 3 %green selected
            primary_stain = 3;
    end

    switch stain_selection(3) %Secondary stain option
        case 1 %dapi selected
            secondary_stain = 1;
            nuclear_list2 = cell(1,number_of_images);
            single_positiveS = zeros(1,number_of_images);
            double_positiveS = zeros(1,number_of_images);
            triple_plus_positiveS = zeros(1,number_of_images);
            stain_negativeS = zeros(1,number_of_images);
            total_number_of_nucleiS = zeros(1,number_of_images);
            fusion_indexS = zeros(1,number_of_images);
            negative_siS = zeros(1,number_of_images);
            coeff_diffS = zeros(1,number_of_images);
        case 2 %red selected
            secondary_stain = 2;
            nuclear_list2 = cell(1,number_of_images);
            single_positiveS = zeros(1,number_of_images);
            double_positiveS = zeros(1,number_of_images);
            triple_plus_positiveS = zeros(1,number_of_images);
            stain_negativeS = zeros(1,number_of_images);
            total_number_of_nucleiS = zeros(1,number_of_images);
            fusion_indexS = zeros(1,number_of_images);
            negative_siS = zeros(1,number_of_images);
            coeff_diffS = zeros(1,number_of_images);
        case 3 %green selected
            secondary_stain = 3;
            nuclear_list2 = cell(1,number_of_images);
            single_positiveS = zeros(1,number_of_images);
            double_positiveS = zeros(1,number_of_images);
            triple_plus_positiveS = zeros(1,number_of_images);
            stain_negativeS = zeros(1,number_of_images);
            total_number_of_nucleiS = zeros(1,number_of_images);
            fusion_indexS = zeros(1,number_of_images);
            negative_siS = zeros(1,number_of_images);
            coeff_diffS = zeros(1,number_of_images);
    end


   if debug == 1
       if number_of_debug_images ~= 0
       number_of_images = number_of_debug_images;
       end
   end


for image_counter = 1:number_of_images

  if debug == 1
    filename1 = image_array{image_counter,1};
    [dummyvar1,file_image_name,dummyvar2] = fileparts(filename1);
  else
    filename1 = image_list(image_counter,:);
    [file_dir,file_image_name,file_image_extension] = fileparts(filename1);
  end

    display(['>>> Processing Image Number ', num2str(image_counter),' of ',num2str(number_of_images)]);
    cd(dir1);
    Ig = uint8(imread(filename1));
    Gaussian_filter = fspecial('gaussian',[5 5],10);
```

```
    if blur_factor >= 1
        for i = 1:blur_factor
            IgD = imfilter(Ig,Gaussian_filter,'same');
        end
    else
        IgD = Ig;
    end


    Image_stage2d      = IgD(:,:,nuclear_stain) - imopen(IgD(:,:,nuclear_stain),strel('disk',round(double
(disk1))));

    Image_stage2d(Image_stage2d >= contrast_threshold1) = 255;%Anything above threshold value is now
totally blue
    Image_stage2d(Image_stage2d <  contrast_threshold1)  = 0;%Anything below threshold value is now
totally black

    [Bd]     = bwboundaries(Image_stage2d,4,'noholes');
    LBD      = length(Bd);

    if plot_or_not ~= 0
    f = figure('Visible','off');
    set(gcf,'InvertHardcopy','off')
    imshow(Image_stage2d.*0,'Border','tight'); hold on;
    end

    clear centroidBlue x_sum image_names

    for k=1:LBD
       boundary = Bd{k};
       bx = boundary(:,1);
       by = boundary(:,2);

       try
       if length(bx)> 2 && length(by) > 2 && length(find(bx == mean(bx))) ~= length(bx) && length(find(by
== mean(by))) ~= length(by)
            y = polygeom(bx,by);
            x_sum(k) = y(1); %Area
            centroidBlue(k,:) = [y(2),y(3)]; %Centroid (X,Y)
            image_names(k) = image_counter;
            if plot_or_not ~= 0
                %Uncomment If/Elseif to modify nuclei color based on size:
                %if x_sum(k) >= 50 && x_sum(k) < 64
                    f =   fill(by,bx,'g');
%                  elseif x_sum(k) >= 10 && x_sum(k) < 50
%                      f =   fill(by,bx,'y');
%                 end
            end
       end
    end
    image_processing_k(image_counter) = LBD;
    end
   if LBD == 0 || isempty(x_sum)
           x_sum = 0;
           centroidBlue = [0,0];
           image_names = 0;
           display('NO NUCLEAR STAIN DETECTED IN IMAGE!')
   end
    area{:,image_counter} = x_sum';
    centroids{:,image_counter} = centroidBlue;
    image_name{:,image_counter} = image_names';
 %%%%%%
 %%%%%%PRIMARY STAIN
 if stain_selection(2) ~= 4 %if primary stain is selected
 Gaussian_filter = fspecial('gaussian',[5 5],10);
    if blur_factorPrimary >= 1
        for i = 1:blur_factorPrimary
            IgP = imfilter(Ig,Gaussian_filter,'same');
        end
    else
            IgP = Ig;
    end
```

```
    Image_stage2r       = IgP(:,:,primary_stain) - imopen(IgP(:,:,primary_stain),strel('disk',round(double
(disk2))));
    Image_stage2r(Image_stage2r >= contrast_threshold2) = 255;%Anything above threshold value is now
totally blue
    Image_stage2r(Image_stage2r <  contrast_threshold2) = 0;

    [Bcolor] = bwboundaries(Image_stage2r,4); BcolorLength = length(Bcolor);
    display(['Number of stained regions found: ', num2str(BcolorLength)]);
    current_centroids = centroids{image_counter};
    centroidY = current_centroids(:,1);
    centroidX = current_centroids(:,2);
    nuclear_container = zeros(length(centroidY),BcolorLength);

    for j=1:BcolorLength
            boundary = Bcolor{j};
            bx = boundary(:,1);
            by = boundary(:,2);
            nuclear_container(:,j) = inpolygon(centroidY,centroidX,bx,by);
            if plot_or_not ~= 0
            fill(by,bx,'r');
            end
    end
    nuclear_list{image_counter} = sum(nuclear_container,2);
    clear k nuclear_container
 end
%%%%%%
%%%%%%SECONDARY STAIN
if stain_selection(3) ~= 4
Gaussian_filter = fspecial('gaussian',[5 5],10);
    if blur_factorSecondary >= 1
        for i = 1:blur_factorSecondary
            IgS = imfilter(Ig,Gaussian_filter,'same');
        end
    else
            IgS = Ig;
    end
    Image_stage2s       = IgS(:,:,secondary_stain) - imopen(IgS(:,:,secondary_stain),strel('disk',round
(double(disk3))));
    Image_stage2s(Image_stage2s >= contrast_threshold3) = 255;%Anything above threshold value is now
totally blue
    Image_stage2s(Image_stage2s <  contrast_threshold3) = 0;

    [BcolorS] = bwboundaries(Image_stage2s,4); BcolorLengthS = length(BcolorS);
    display(['Number of stained regions found: ', num2str(BcolorLengthS)]);

    if exist('centroidY') == 0 && exist('centroidX') == 0
        current_centroids = centroids{image_counter};
        centroidY = current_centroids(:,1);
        centroidX = current_centroids(:,2);
    end
    nuclear_container2 = zeros(length(centroidY),BcolorLengthS);
    for j=1:BcolorLengthS
            boundary = BcolorS{j};
            bxS = boundary(:,1);
            byS = boundary(:,2);
            nuclear_container2(:,j) = inpolygon(centroidY,centroidX,bxS,byS);
            if plot_or_not ~= 0
            fill(by,bx,'g');
            end
    end
    nuclear_list2{image_counter} = sum(nuclear_container2,2);
    clear k nuclear_container2 Ig
end

    if plot_or_not ~= 0
        for k=1:LBD
       boundary = Bd{k};
       bx = boundary(:,1);
       by = boundary(:,2);
       f =   fill(by,bx,'b');
       set(f,'edgecolor','none')
        end
    end
if stain_selection(2) ~= 4
[sp,dp,tpp,sn,tnn,fi,nsi,cdf,negative_list] = primary_count(image_counter,area,first_peak_center,
```

```
first_peak_lower,first_peak_upper,nuclear_list);
single_positive(image_counter) = sp(image_counter);
double_positive(image_counter) = dp(image_counter);
triple_plus_positive(image_counter) = tpp(image_counter);
stain_negative(image_counter) = sn(image_counter);
total_number_of_nuclei(image_counter) = tnn(image_counter);
fusion_index(image_counter) = fi(image_counter);
negative_si(image_counter) = nsi(image_counter);
coeff_diff(image_counter) = cdf(image_counter);
end

if stain_selection(3) ~= 4
[spS,dpS,tppS,snS,tnnS,fiS,nsiS,cdfS] = secondary_count(image_counter,area,first_peak_center,↙
first_peak_lower,first_peak_upper,nuclear_list2);
single_positiveS(image_counter) = spS(image_counter);
double_positiveS(image_counter) = dpS(image_counter);
triple_plus_positiveS(image_counter) = tppS(image_counter);
stain_negativeS(image_counter) = snS(image_counter);
total_number_of_nucleiS(image_counter) = tnnS(image_counter);
fusion_indexS(image_counter) = fiS(image_counter);
negative_siS(image_counter) = nsiS(image_counter);
coeff_diffS(image_counter) = cdfS(image_counter);
end
negative_nuclei_indecies = find(negative_list > 0);
    if plot_or_not ~= 0
        cdata = hardcopy(gcf, '-Dzbuffer', '-r0');
        close gcf
        image = cdata;
        cd(dir3);
        if trace_directory_success == 1
            try
                imwrite(image,fullfile(dir3,['Trace Image_' file_image_name '.tif']),'tif','Resolution',↙
300)
                display(['Trace image ' num2str(image_counter) ' saved.']);
            catch
                display('Directory has been created, but there is an error in writing the images to↙
disk');
            end
        else
                display(['Directory not created, trace image NOT saved.']);
        end
    end
end
display('Counting Analysis Finished!');

if stain_selection(2) ~= 4 && stain_selection(3) == 4 %Primary Stain ON, Secondary OFF
display('Primary ON, Secondary OFF')
    if data_save == 1
         titles = ['Image name,' '# of nuclei,' '# of Stain Negative,' '# of Single Cells,' '# of double↙
fusions,' '# of triple+ fusions,' 'fusion index,' 'negative stain index,' 'differentiation coeff'];
         [success,fopendir] = save_data_to_file(debug,c,titles,number_of_images,dir1,image_list,↙
total_number_of_nuclei,stain_negative,single_positive,double_positive,triple_plus_positive,fusion_index,↙
negative_si,coeff_diff);
         if success == 1
              display(['Data saved to file: ' fopendir]);
         else
              display('Data not saved')
         end
    else
         display('No data has been saved')
    end
end

if stain_selection(3) ~= 4 && stain_selection(2) == 4 %Primary OFF, Secondary ON
display('Primary OFF, Secondary ON')
    if data_save == 1
    titles = ['Image name,' '# of nuclei,' '# of Stain Negative Secondary,' '# of Single Cells Secondary,'↙
'# of double fusions Secondary,' '# of triple+ fusions Secondary,' 'fusion index Secondary,' 'negative↙
stain index Secondary,' 'differentiation coeff Secondary'];
    [success,fopendir] = save_data_to_file(debug,c,titles,number_of_images,dir1,image_list,↙
total_number_of_nucleiS,stain_negativeS,single_positiveS,double_positiveS,triple_plus_positiveS,↙
fusion_indexS,negative_siS,coeff_diffS);
    if success == 1
         display(['Data saved to file: ' fopendir]);
    else
```

```
        display('Data not saved')
    end
else
    display('No data has been saved')
end
end

if stain_selection(3) ~= 4 && stain_selection(2) ~= 4 %Primary ON, Secondary ON (BOTH ON)
display('Primary ON, Secondary ON')
    if data_save == 1
    titles = ['Image name,' '# of nuclei,' '# of Stain Negative,' '# of Single Cells,' '# of double↙
fusions,' '# of triple+ fusions,' 'fusion index,' 'negative stain index,' 'differentiation coeff,' '# of↙
Stain Negative Secondary,' '# of Single Cells Secondary,' '# of double fusions Secondary,' '# of triple+↙
fusions Secondary,' 'fusion index Secondary,' 'negative stain index Secondary,' 'differentiation coeff↙
Secondary'];
    [success,fopendir] = save_data_to_file(debug,c,titles,number_of_images,dir1,image_list,↙
total_number_of_nuclei,stain_negative,single_positive,double_positive,triple_plus_positive,fusion_index,↙
negative_si,coeff_diff,stain_negativeS,single_positiveS,double_positiveS,triple_plus_positiveS,↙
fusion_indexS,negative_siS,coeff_diffS);
    if success == 1
        display(['Data saved to file: ' fopendir]);
    else
        display('Data not saved')
    end
else
    display('No data has been saved')
end
end

if stain_selection(3) == 4 && stain_selection(2) == 4 %Primary OFF, Secondary OFF (BOTH OFF)
if data_save == 1
    titles = ['Image name,' '# of nuclei'];
    [success,fopendir] = save_data_to_file(debug,c,titles,number_of_images,dir1,image_list,↙
total_number_of_nuclei);
    if success == 1
        display(['Nuclear Counting Data saved to file: ' fopendir]);
    else
        display('Nuclear Counting Data not saved')
    end
else
    display('No data has been saved, No primary or secondary stains selected')
end
end
```
