# Supplementary material for: Satellite cell heterogeneity revealed by G-Tool, an open algorithm to quantify myogenesis through colony-forming assays
Source: Skelet Muscle. 2012 Jun 15;2:13. doi: 10.1186/2044-5040-2-13 (PMC3439689; doi:10.1186/2044-5040-2-13)
Supplement: Additional file 1 — G-Tool Source Code. Java and MATLAB Source Codes are included. [file 2044-5040-2-13-S1.zip › G-Tool Sourcecode and PDF files/PDF files of code/MATLAB - Algorithm/polygeom.pdf]

```
function [ geom, iner, cpmo ] = polygeom( x, y )
% Copyright (c) 1998, H.J. Sommer
% All rights reserved.
%
% Redistribution and use in source and binary forms, with or without
% modification, are permitted provided that the following conditions are
% met:
%
%     * Redistributions of source code must retain the above copyright
%       notice, this list of conditions and the following disclaimer.
%     * Redistributions in binary form must reproduce the above copyright
%       notice, this list of conditions and the following disclaimer in
%       the documentation and/or other materials provided with the distribution
%     * Neither the name of the Penn State University nor the names
%       of its contributors may be used to endorse or promote products derived
%       from this software without specific prior written permission.
%
% THIS SOFTWARE IS PROVIDED BY THE COPYRIGHT HOLDERS AND CONTRIBUTORS "AS IS"
% AND ANY EXPRESS OR IMPLIED WARRANTIES, INCLUDING, BUT NOT LIMITED TO, THE
% IMPLIED WARRANTIES OF MERCHANTABILITY AND FITNESS FOR A PARTICULAR PURPOSE
% ARE DISCLAIMED. IN NO EVENT SHALL THE COPYRIGHT OWNER OR CONTRIBUTORS BE
% LIABLE FOR ANY DIRECT, INDIRECT, INCIDENTAL, SPECIAL, EXEMPLARY, OR
% CONSEQUENTIAL DAMAGES (INCLUDING, BUT NOT LIMITED TO, PROCUREMENT OF
% SUBSTITUTE GOODS OR SERVICES; LOSS OF USE, DATA, OR PROFITS; OR BUSINESS
% INTERRUPTION) HOWEVER CAUSED AND ON ANY THEORY OF LIABILITY, WHETHER IN
% CONTRACT, STRICT LIABILITY, OR TORT (INCLUDING NEGLIGENCE OR OTHERWISE)
% ARISING IN ANY WAY OUT OF THE USE OF THIS SOFTWARE, EVEN IF ADVISED OF THE
% POSSIBILITY OF SUCH DAMAGE.

%POLYGEOM Geometry of a planar polygon
%
%   POLYGEOM( X, Y ) returns area, X centroid,
%   Y centroid and perimeter for the planar polygon
%   specified by vertices in vectors X and Y.
%
%   [ GEOM, INER, CPMO ] = POLYGEOM( X, Y ) returns
%   area, centroid, perimeter and area moments of
%   inertia for the polygon.
%   GEOM = [ area   X_cen  Y_cen  perimeter ]
%   INER = [ Ixx    Iyy    Ixy    Iuu    Ivv    Iuv ]
%     u,v are centroidal axes parallel to x,y axes.
%   CPMO = [ I1     ang1   I2     ang2   J ]
%     I1,I2 are centroidal principal moments about axes
%         at angles ang1,ang2.
%     ang1 and ang2 are in radians.
%     J is centroidal polar moment.  J = I1 + I2 = Iuu + Ivv

% H.J. Sommer III - 02.05.14 - tested under MATLAB v5.2
%
% sample data
% x = [ 2.000  0.500  4.830  6.330 ]';
% y = [ 4.000  6.598  9.098  6.500 ]';
% 3x5 test rectangle with long axis at 30 degrees
% area=15, x_cen=3.415, y_cen=6.549, perimeter=16
% Ixx=659.561, Iyy=201.173, Ixy=344.117
% Iuu=16.249, Ivv=26.247, Iuv=8.660
% I1=11.249, ang1=30deg, I2=31.247, ang2=120deg, J=42.496
%
% H.J. Sommer III, Ph.D., Professor of Mechanical Engineering, 337 Leonhard Bldg
% The Pennsylvania State University, University Park, PA  16802
% (814)863-8997  FAX (814)865-9693  hjs1@psu.edu  www.me.psu.edu/sommer/

% begin function POLYGEOM

% check if inputs are same size
if ~isequal( size(x), size(y) ),
  error( 'X and Y must be the same size');
end

% number of vertices
[ x, ns ] = shiftdim( x );
[ y, ns ] = shiftdim( y );
[ n, c ] = size( x );

% temporarily shift data to mean of vertices for improved accuracy
```

```
xm = mean(x);
ym = mean(y);
x = x - xm*ones(n,1);
y = y - ym*ones(n,1);

% delta x and delta y
dx = x( [ 2:n 1 ] ) - x;
dy = y( [ 2:n 1 ] ) - y;

% summations for CW boundary integrals
A = sum( y.*dx - x.*dy )/2;
Axc = sum( 6*x.*y.*dx -3*x.*x.*dy +3*y.*dx.*dx +dx.*dx.*dy )/12;
Ayc = sum( 3*y.*y.*dx -6*x.*y.*dy -3*x.*dy.*dy -dx.*dy.*dy )/12;
Ixx = sum( 2*y.*y.*y.*dx -6*x.*y.*y.*dy -6*x.*y.*dy.*dy ...
          -2*x.*dy.*dy.*dy -2*y.*dx.*dy.*dy -dx.*dy.*dy.*dy )/12;
Iyy = sum( 6*x.*x.*y.*dx -2*x.*x.*x.*dy +6*x.*y.*dx.*dx ...
          +2*y.*dx.*dx.*dx +2*x.*dx.*dx.*dy +dx.*dx.*dx.*dy )/12;
Ixy = sum( 6*x.*y.*y.*dx -6*x.*x.*y.*dy +3*y.*y.*dx.*dx ...
          -3*x.*x.*dy.*dy +2*y.*dx.*dx.*dy -2*x.*dx.*dy.*dy )/24;
P = sum( sqrt( dx.*dx +dy.*dy ) );

% check for CCW versus CW boundary
if A < 0,
  A = -A;
  Axc = -Axc;
  Ayc = -Ayc;
  Ixx = -Ixx;
  Iyy = -Iyy;
  Ixy = -Ixy;
end

% centroidal moments
xc = Axc / A;
yc = Ayc / A;
Iuu = Ixx - A*yc*yc;
Ivv = Iyy - A*xc*xc;
Iuv = Ixy - A*xc*yc;
J = Iuu + Ivv;

% replace mean of vertices
x_cen = xc + xm;
y_cen = yc + ym;
Ixx = Iuu + A*y_cen*y_cen;
Iyy = Ivv + A*x_cen*x_cen;
Ixy = Iuv + A*x_cen*y_cen;

% principal moments and orientation
I = [ Iuu  -Iuv ;
     -Iuv   Ivv ];
[ eig_vec, eig_val ] = eig(I);
I1 = eig_val(1,1);
I2 = eig_val(2,2);
ang1 = atan2( eig_vec(2,1), eig_vec(1,1) );
ang2 = atan2( eig_vec(2,2), eig_vec(1,2) );

% return values
geom = [ A  x_cen  y_cen  P ];
iner = [ Ixx  Iyy  Ixy  Iuu  Ivv  Iuv ];
cpmo = [ I1  ang1  I2  ang2  J ];

% end of function POLYGEOM
```
