# Supplementary material for: Satellite cell heterogeneity revealed by G-Tool, an open algorithm to quantify myogenesis through colony-forming assays
Source: Skelet Muscle. 2012 Jun 15;2:13. doi: 10.1186/2044-5040-2-13 (PMC3439689; doi:10.1186/2044-5040-2-13)
Supplement: Additional file 1 — G-Tool Source Code. Java and MATLAB Source Codes are included. [file 2044-5040-2-13-S1.zip › G-Tool Sourcecode and PDF files/PDF files of code/MATLAB - Algorithm/save_data_to_file.pdf]

```
function [success,fopendir] = save_data_to_file(debug,c,titles,number_of_images,save_directory,image_list,
total_number_of_nuclei,varargin)
%   This file is part of GTOOL. AUTHOR: JOSEPH IPPOLITO, THE UNIVERSITY
%   OF MINNESOTA. GTOOL is free software: you can redistribute it
%   and/or modify
%   it under the terms of the GNU General Public License as published
%   by the Free Software Foundation, either version 3 of the License, or
%   (at your option) any later version.
%   GTOOL is distributed in the hope that it will be useful,
%   but WITHOUT ANY WARRANTY; without even the implied warranty of
%   MERCHANTABILITY or FITNESS FOR A PARTICULAR PURPOSE. SEE THE GNU
%   GENERAL PUBLIC LISCENCE FOR MORE DETAILS.
%   You should have received a copy of the GNU General Public License
%   along with GTOOL. If not see see <http://www.gnu.org/licenses/>.
if nargin == 14
stain_negative = varargin{1};
single_positive = varargin{2};
double_positive = varargin{3};
triple_plus_positive = varargin{4};
fusion_index = varargin{5};
negative_si = varargin{6};
coeff_diff    = varargin{7};
end

if nargin == 21
stain_negative = varargin{1};
single_positive = varargin{2};
double_positive = varargin{3};
triple_plus_positive = varargin{4};
fusion_index = varargin{5};
negative_si = varargin{6};
coeff_diff    = varargin{7};
stain_negativeS = varargin{8};
single_positiveS = varargin{9};
double_positiveS = varargin{10};
triple_plus_positiveS = varargin{11};
fusion_indexS = varargin{12};
negative_siS = varargin{13};
coeff_diffS = varargin{14};
end

datafilename = ['Data_' num2str(c(1)) '-' num2str(c(2)) '-' num2str(c(3)) '-' num2str(c(4)) '-' num2str(c
(5)) '.csv'];
fopendir = fullfile(save_directory,datafilename);

[fid,message] = fopen(fopendir,'w');


if debug == 1
        image_array = cell(length(image_list),1);

    for i = 1:length(image_list)
        image_array{i,1} = image_list(i).name;
    end
 end



if isempty(message)
    fprintf(fid, titles);
    for i = 1:number_of_images



        if debug == 1
             filename1 = image_array{i,1};
             [dummyvar1,file_image_name,dummyvar2] = fileparts(filename1);
        else
             filename1 = image_list(i,:);
            [file_dir,file_image_name,file_image_extension] = fileparts(filename1);
        end

    if nargin == 7
        y = [file_image_name ',' num2str(total_number_of_nuclei(i))];
    elseif nargin == 14
```

```
        y = [file_image_name ',' num2str(total_number_of_nuclei(i)) ',' num2str(stain_negative(i)) ','↙
num2str(single_positive(i)) ',' num2str(double_positive(i)) ',' num2str(triple_plus_positive(i)) ','↙
num2str(fusion_index(i)) ',' num2str(negative_si(i)) ',' num2str(coeff_diff(i)) ];
    elseif nargin == 21
        y = [file_image_name ',' num2str(total_number_of_nuclei(i)) ',' num2str(stain_negative(i)) ','↙
num2str(single_positive(i)) ',' num2str(double_positive(i)) ',' num2str(triple_plus_positive(i)) ','↙
num2str(fusion_index(i)) ',' num2str(negative_si(i)) ',' num2str(coeff_diff(i)) ',' num2str↙
(stain_negativeS(i)) ',' num2str(single_positiveS(i)) ',' num2str(double_positiveS(i)) ',' num2str↙
(triple_plus_positiveS(i)) ',' num2str(fusion_indexS(i)) ',' num2str(negative_siS(i)) ',' num2str↙
(coeff_diffS(i))];
    end


        fprintf(fid, '\r\n %s', y');
    end
    fclose(fid);
    success = 1;
else
    display(message)
    success = 0;
end
```
