# Supplementary material for: Satellite cell heterogeneity revealed by G-Tool, an open algorithm to quantify myogenesis through colony-forming assays
Source: Skelet Muscle. 2012 Jun 15;2:13. doi: 10.1186/2044-5040-2-13 (PMC3439689; doi:10.1186/2044-5040-2-13)
Supplement: Additional file 1 — G-Tool Source Code. Java and MATLAB Source Codes are included. [file 2044-5040-2-13-S1.zip › G-Tool Sourcecode and PDF files/PDF files of code/MATLAB - Algorithm/secondary_count.pdf]

```
function [single_positive,double_positive,triple_plus_positive,stain_negative,total_number_of_nuclei,↙
fusion_index,negative_si,coeff_diff] = secondary_count(image_counter,area,first_peak_center,↙
first_peak_lower,first_peak_upper,nuclear_list)
%   This file is part of GTOOL. AUTHOR: JOSEPH IPPOLITO, THE UNIVERSITY
%   OF MINNESOTA. GTOOL is free software: you can redistribute it
%   and/or modify
%   it under the terms of the GNU General Public License as published
%   by the Free Software Foundation, either version 3 of the License, or
%   (at your option) any later version.
%   GTOOL is distributed in the hope that it will be useful,
%   but WITHOUT ANY WARRANTY; without even the implied warranty of
%   MERCHANTABILITY or FITNESS FOR A PARTICULAR PURPOSE. SEE THE GNU
%   GENERAL PUBLIC LISCENCE FOR MORE DETAILS.
%   You should have received a copy of the GNU General Public License
%   along with GTOOL. If not see see <http://www.gnu.org/licenses/>.

    f = area{image_counter}./first_peak_center;
    f(f<first_peak_lower) = 0;
    f(f>=first_peak_lower & f<=first_peak_upper) = 1;
    second_peak_limit = abs(2*first_peak_upper - 1);
    %This now uses characteristics of the 1st peak to determine the cutoff
    %for doubles. It appears to be more accurate this way, rather than
    %using 2 (double the first peak) as a limit.
        if second_peak_limit == 0
            second_peak_limit = 2;
        end
    f_list{image_counter} = f;

    positive_list = nuclear_list{image_counter}.*f_list{image_counter};
    negative_list = (1-nuclear_list{image_counter}).*f_list{image_counter};

    single_positive(image_counter)      = sum(positive_list(positive_list == 1));
    double_positive(image_counter)      = sum(positive_list(positive_list > first_peak_upper &↙
positive_list <= 2));
    triple_plus_positive(image_counter) = sum(positive_list(positive_list > second_peak_limit));

    stain_negative(image_counter)         = sum(sum(negative_list));
    total_number_of_nuclei(image_counter) = sum(f_list{image_counter},1);

    fusion_index(image_counter) =  triple_plus_positive(image_counter)/total_number_of_nuclei↙
(image_counter);
    negative_si(image_counter)  =  stain_negative(image_counter)/total_number_of_nuclei(image_counter);
    coeff_diff(image_counter)   = (single_positive(image_counter)+double_positive(image_counter)↙
+triple_plus_positive(image_counter))/total_number_of_nuclei(image_counter);
```
