# Supplementary material for: Satellite cell heterogeneity revealed by G-Tool, an open algorithm to quantify myogenesis through colony-forming assays
Source: Skelet Muscle. 2012 Jun 15;2:13. doi: 10.1186/2044-5040-2-13 (PMC3439689; doi:10.1186/2044-5040-2-13)
Supplement: Additional file 1 — G-Tool Source Code. Java and MATLAB Source Codes are included. [file 2044-5040-2-13-S1.zip › G-Tool Sourcecode and PDF files/PDF files of code/MATLAB - Algorithm/silent_running.pdf]

```
function [image] = silent_running(plot_or_not)
%   This file is part of GTOOL. AUTHOR: JOSEPH IPPOLITO, THE UNIVERSITY
%   OF MINNESOTA. GTOOL is free software: you can redistribute it
%   and/or modify
%   it under the terms of the GNU General Public License as published
%   by the Free Software Foundation, either version 3 of the License, or
%   (at your option) any later version.
%   GTOOL is distributed in the hope that it will be useful,
%   but WITHOUT ANY WARRANTY; without even the implied warranty of
%   MERCHANTABILITY or FITNESS FOR A PARTICULAR PURPOSE. SEE THE GNU
%   GENERAL PUBLIC LISCENCE FOR MORE DETAILS.
%   You should have received a copy of the GNU General Public License
%   along with GTOOL. If not see see <http://www.gnu.org/licenses/>.
   if plot_or_not ~= 2
   cdata = hardcopy(gcf, '-Dzbuffer', '-r0');
   close gcf
   image = cdata;
   else
   image = [1;1;1];
   end

end
```
